# Different Profiles of Spatial Navigation Deficits In Alzheimer’s Disease Biomarker-Positive Versus Biomarker-Negative Older Adults With Amnestic Mild Cognitive Impairment

**DOI:** 10.3389/fnagi.2022.886778

**Published:** 2022-06-02

**Authors:** Martina Laczó, Lukas Martinkovic, Ondrej Lerch, Jan M. Wiener, Jana Kalinova, Veronika Matuskova, Zuzana Nedelska, Martin Vyhnalek, Jakub Hort, Jan Laczó

**Affiliations:** ^1^Memory Clinic, Department of Neurology, Charles University, Second Faculty of Medicine and Motol University Hospital, Prague, Czechia; ^2^International Clinical Research Center, St. Anne’s University Hospital Brno, Brno, Czechia; ^3^Department of Psychology, Ageing and Dementia Research Centre, Bournemouth University, Poole, United Kingdom

**Keywords:** egocentric navigation, allocentric navigation, hippocampus, entorhinal cortex, precuneus, retrosplenial cortex, neurodegeneration, tauopathies

## Abstract

**Background:**

Spatial navigation impairment is a promising cognitive marker of Alzheimer’s disease (AD) that can reflect the underlying pathology.

**Objectives:**

We assessed spatial navigation performance in AD biomarker positive older adults with amnestic mild cognitive impairment (AD aMCI) vs. those AD biomarker negative (non-AD aMCI), and examined associations between navigation performance, MRI measures of brain atrophy, and cerebrospinal fluid (CSF) biomarkers.

**Methods:**

A total of 122 participants with AD aMCI (*n* = 33), non-AD aMCI (*n* = 31), mild AD dementia (*n* = 28), and 30 cognitively normal older adults (CN) underwent cognitive assessment, brain MRI (*n* = 100 had high-quality images for volumetric analysis) and three virtual navigation tasks focused on route learning (body-centered navigation), wayfinding (world-centered navigation) and perspective taking/wayfinding. Cognitively impaired participants underwent CSF biomarker assessment [amyloid-β_1–42_, total tau, and phosphorylated tau_181_ (p-tau_181_)] and amyloid PET imaging (*n* = 47 and *n* = 45, respectively), with a subset having both (*n* = 19).

**Results:**

In route learning, AD aMCI performed worse than non-AD aMCI (*p* < 0.001), who performed similarly to CN. In wayfinding, aMCI participants performed worse than CN (both *p* ≤ 0.009) and AD aMCI performed worse than non-AD aMCI in the second task session (*p* = 0.032). In perspective taking/wayfinding, aMCI participants performed worse than CN (both *p* ≤ 0.001). AD aMCI and non-AD aMCI did not differ in conventional cognitive tests. Route learning was associated with parietal thickness and amyloid-β_1–42_, wayfinding was associated with posterior medial temporal lobe (MTL) volume and p-tau_181_ and perspective taking/wayfinding was correlated with MRI measures of several brain regions and all CSF biomarkers.

**Conclusion:**

AD biomarker positive and negative older adults with aMCI had different profiles of spatial navigation deficits that were associated with posterior MTL and parietal atrophy and reflected AD pathology.

## Introduction

Alzheimer’s disease (AD) is the most common neurodegenerative disease (Barker et al., 2002). AD forms a continuum from the preclinical stage through mild cognitive impairment (MCI) to dementia (Jack et al., 2018). AD is associated with extracellular aggregation of amyloid-β plaques (Thal et al., 2002), intracellular accumulation of abnormally phosphorylated tau protein (Braak and Braak, 1995), progressive neuronal loss (neurodegeneration; Miller et al., 2013) and cognitive impairment (Green et al., 2000). Amyloid-β accumulation first emerges in the neocortical regions, and early affects the posterior-midline regions such as the precuneus and posterior cingulate cortex, including the retrosplenial cortex (RSC; Sojkova et al., 2011; Palmqvist et al., 2017). Tau pathology first emerges in the transentorhinal cortex [the region between the anterolateral entorhinal cortex (alEC) and the perirhinal cortex] in Braak stage I and spreads to the posteromedial entorhinal cortex (pmEC) and the hippocampus (Braak and Braak, 1995) in Braak stages II and III, respectively. The pathology then spreads to the posterior cortical regions (Braak stage IV) in the early clinical stages (i.e., MCI), and finally to the entire neocortex (Braak stages V and VI) in the dementia stage (Braak and Braak, 1991). Neurodegeneration and brain atrophy essentially parallel the pattern of distribution and propagation of tau pathology (Whitwell et al., 2007) but not amyloid-β deposition (Josephs et al., 2008) in AD. The regions early affected by AD pathology are essential for spatial navigation (Hartley et al., 2003; Blanch et al., 2004; Howett et al., 2019; Schöberl et al., 2020). Spatial navigation is a complex cognitive process of determining and updating one’s position and orientation in the environment (Wolbers and Hegarty, 2010). Spatial navigation deficits may thus be one of the earliest cognitive markers of AD, and the assessment of spatial navigation may aid the early diagnosis of AD (Coughlan et al., 2018).

Successful spatial navigation requires a flexible combination of various navigation strategies. When navigating the environment, navigators can remember the traveled route in relation to their own bodies. This navigation strategy is referred to as route learning (i.e., body-centered, egocentric navigation). When learning routes, navigators can encode the sequence of body movements at decision points (e.g., right, left, straight) or form associations between direction changes and specific proximal landmarks (e.g., “Turn left at the shop”; Waller and Lippa, 2007). The posterior parietal cortex (DeIpolyi et al., 2007; Ruotolo et al., 2019), precuneus (Weniger et al., 2011; Saj et al., 2014), and the caudate nucleus (Iglói et al., 2010) play an important role in route learning (Hartley et al., 2003; Blanch et al., 2004). Alternatively, navigators can create an internal representation of the environment (i.e., “cognitive map”) by encoding positions of distant landmarks relative to each other and relative to specific locations regardless of the navigators’ positions. This navigation strategy is referred to as wayfinding (i.e., world-centered, allocentric navigation). The medial temporal lobe (MTL), especially the hippocampus, which is strongly interconnected with the entorhinal cortex (EC; Cholvin et al., 2021), is important for wayfinding (Maguire et al., 1998). The right hippocampus is more strongly associated with wayfinding than the left hippocampus (Maguire et al., 1998; Nedelska et al., 2012; Laczó et al., 2017). The hippocampal and EC subregions show different functional specialization along the anterior-posterior axis, with the posterior regions being more relevant for wayfinding (Doeller et al., 2008). Specifically, the posterior hippocampus (i.e., the body and the tail) is involved in the creation and use of cognitive maps (Schinazi et al., 2013) and in supporting fine-grained spatial representations (Brunec et al., 2018), while the anterior hippocampus (i.e., the head) is involved in responding to novelty (Doeller et al., 2008) and navigational planning (Xu et al., 2010). Within the EC, the pmEC is involved in spatial information processing (Reagh and Yassa, 2014), world-centered direction computations (Chadwick et al., 2015), and fine-grained spatial representations (Evensmoen et al., 2015), while the alEC is involved in object information processing (Reagh et al., 2018) and encoding of distances between locations related to landmarks (Chen et al., 2019). For successful navigation, it is essential to integrate body-centered and world-centered spatial information (i.e., reference frame translation) and use landmarks for directional orientation, both of which are supported by the RSC (Auger et al., 2012; Clark et al., 2018). Navigators can imagine scenes or parts of the environment from different perspectives during navigation, which is referred to as perspective taking (Marková et al., 2015). The parietal and temporal cortex and the MTL play an important role in this process (Zacks and Michelon, 2005; Lambrey et al., 2008).

Spatial navigation deteriorates as AD progresses along the AD continuum (Hort et al., 2007; Allison et al., 2016; Levine et al., 2020). Older adults with mild AD dementia are severely impaired in route learning and wayfinding, both in real space (DeIpolyi et al., 2007; Hort et al., 2007) and in the virtual environment (Cushman et al., 2008; Laczó et al., 2010). They also show deficits in switching between the two spatial strategies (Morganti et al., 2013) and in perspective taking (Marková et al., 2015). Older adults with amnestic MCI (aMCI) frequently show deficits in route learning and wayfinding, both in real space (DeIpolyi et al., 2007; Laczó et al., 2009) and in the virtual environment (Weniger et al., 2011; Laczó et al., 2012). Perspective taking deficits have also been found in individuals with aMCI (Marková et al., 2015; Laczó et al., 2021a). However, these studies did not use biomarkers to confirm that AD was the cause of aMCI. The recent biomarker studies indicated that aMCI individuals with AD (AD aMCI) compared to cognitively normal (CN) older adults are impaired in wayfinding (Parizkova et al., 2018; Schöberl et al., 2020) and route learning (Schöberl et al., 2020) in real space and in recognition of the environment from a shifted viewpoint (Chan et al., 2016). One study suggested that older adults with preclinical AD [i.e., CN individuals with low cerebrospinal fluid (CSF) amyloid-β_1–42_ levels] have deficits in wayfinding but not route learning in the virtual environment (Allison et al., 2016). A recent study from the same research group replicated the findings of wayfinding deficits in preclinical AD and suggested that lower amyloid-β_1–42_ and higher p-tau_181_ in CSF are associated with worse wayfinding performance in CN older adults (Allison et al., 2019). Together, these studies suggest that wayfinding deficits may already be present in preclinical AD, whereas route learning deficits first occur in the early clinical stages of AD.

In the effort to early diagnose AD, it is important to differentiate AD aMCI from those with other causes of aMCI. However, so far only a few studies addressed this topic. One recent study indicated that individuals with aMCI and amyloid-β positivity on positron emission tomography (PET) and in CSF show worse route learning and wayfinding in a complex real environment than those with negative amyloid-β biomarkers (Schöberl et al., 2020). It is worth noting that in contrast to aMCI individuals with amyloid-β positivity, those with negative amyloid-β biomarkers were impaired only in wayfinding and had similar performance to CN older adults in route learning (Schöberl et al., 2020). Similarly, route learning/body-centered navigation performance as opposed to wayfinding/world-centered navigation performance was shown to reliably discriminate cognitively impaired older adults with AD from those with other neurodegenerative diseases (Tu et al., 2017). Together, these studies suggest that route learning tasks have a greater potential to reveal AD-specific navigational deficits compared to wayfinding tasks. A recent study compared older adults with MCI and positive AD biomarkers in CSF to those with AD negative biomarkers. The former group showed less accurate spatial navigation in an immersive virtual reality task in which participants had to rely on self-motion cues (i.e., the path integration task; Howett et al., 2019). It has also been shown that older adults with aMCI and positive CSF AD biomarkers were impaired in recognizing environments from a shifted viewpoint compared to those with negative biomarkers (Chan et al., 2016).

The previous studies indicated that spatial navigation may be a promising diagnostic tool to differentiate individuals with AD aMCI from aMCI individuals with negative AD biomarkers (non-AD aMCI; Howett et al., 2019; Schöberl et al., 2020). However, spatial navigation tests in real space and those in virtual environments that require movement in the real world are not optimal for everyday clinical practice because they are difficult to administer given space constraints in clinical settings and requirements of additional equipment. An ideal spatial navigation assessment should be ecologically valid, easy to administer and explain, and one in which participants can move virtually in a realistic-looking complex environment mimicking the real-life navigation (Diersch and Wolbers, 2019). The Navigation Test Suite^1^, has been developed to meet these requirements (Wiener et al., 2020). It is performed on a computer screen, takes place in a computer-generated realistic-looking virtual city, provides closely controlled testing conditions, and enables manipulation of navigational parameters, such as landmark availability and navigation complexity. The Test Suite has been designed to evaluate various spatial navigation abilities and consists of three tasks: a Route-repetition task assessing route learning (body-centered navigation), a Route-retracing task assessing wayfinding (world-centered navigation), and a Directional-approach task assessing perspective taking and wayfinding (Wiener et al., 2013; de Condappa and Wiener, 2016). Our previous studies showed that this test is well tolerated by CN (Wiener et al., 2020; Laczó et al., 2021a) and cognitively impaired older adults (Laczó et al., 2021a), and reliably detects spatial navigation deficits in individuals with aMCI and mild AD dementia (Laczó et al., 2021a). However, the potential of this test to differentiate individuals with AD aMCI from those with non-AD aMCI, and associations of navigation performance with magnetic resonance imaging (MRI) measures of brain atrophy and AD biomarkers have not been investigated.

In this study, we built on our previous research using the Navigation Test Suite (Wiener et al., 2020; Laczó et al., 2021a) and aimed to assess: (1) the differences in spatial navigation performance in the specific tasks tested between the participants with AD aMCI, non-AD aMCI, mild AD dementia and CN; (2) the associations of spatial navigation performance with MRI measures of atrophy in the specific MTL, cortical and subcortical regions and; (3) the associations of spatial navigation performance with AD biomarkers in CSF, and the role of regional brain atrophy in these associations.

First, we hypothesized that participants with AD aMCI would show worse spatial navigation performance than those with non-AD aMCI in all three navigation tasks. Based on previous findings (Tu et al., 2017; Schöberl et al., 2020), we assumed that the largest differences between AD aMCI and non-AD aMCI participants would be observed in route learning. AD aMCI participants would perform worse than CN participants and similar to participants with mild AD dementia in all three tasks. Participants with non-AD aMCI would perform similarly to CN participants in route learning and worse than CN participants in wayfinding and perspective taking/wayfinding. Second, we hypothesized that more pronounced regional brain atrophy would be associated with worse spatial navigation performance. Specifically, atrophy of the parietal regions including the precuneus and posterior parietal cortex would be preferentially associated with worse route learning performance. Atrophy of the hippocampus and EC, especially their posterior subregions, would be preferentially associated with worse wayfinding performance. Based on previous findings (Nedelska et al., 2012; Laczó et al., 2017), we assumed that the association with wayfinding would be stronger for the right hippocampus than for the left. Atrophy of the hippocampus and EC, especially their posterior subregions, as well as parietal regions would be associated with worse perspective taking/wayfinding performance. Given that the integration of body-centered and world-centered spatial information is required for this task (Wiener et al., 2020), the association with atrophy of the isthmus cingulate/RSC would also be expected. Third, we hypothesized that lower levels of amyloid-β_1–42_ in CSF would be more strongly associated with worse route learning performance given the early and predominant amyloid-β accumulation in the parietal cortex (Sojkova et al., 2011; Palmqvist et al., 2017), the key region for route learning (DeIpolyi et al., 2007; Weniger et al., 2011; Saj et al., 2014; Ruotolo et al., 2019). Higher levels of phosphorylated tau_181_ (p-tau_181_) in CSF would be more strongly associated with worse wayfinding performance given the early and predominant accumulation of tau pathology in the MTL (Braak and Braak, 1991, 1995; i.e., the hippocampus and EC), the key region for wayfinding (Nedelska et al., 2012; Howard et al., 2014; Chen et al., 2019). Levels of amyloid-β_1–42_ and p-tau_181_ in CSF would be associated with perspective taking/wayfinding performance given the early and predominant accumulation of amyloid-β and tau pathology in the parietal cortex and MTL, respectively, the key regions for perspective taking (Zacks and Michelon, 2005; Lambrey et al., 2008). We also hypothesized that the association between CSF amyloid-β_1–42_ levels and performance in the route learning and perspective taking/wayfinding tasks would not be mediated by brain atrophy, as it has been shown that amyloid-β accumulation is not directly linked to regional atrophy (Josephs et al., 2008). Brain atrophy would mediate the association between CSF p-tau_181_ levels and performance in the wayfinding and perspective taking/wayfinding tasks, given that tau pathology was shown to be related to region-specific neurodegeneration (Whitwell et al., 2007).

## Methods

### Participants

#### Recruitment and Inclusion Criteria

A total of 122 participants were included in the study. The participants were recruited from the Czech Brain Aging Study (CBAS) cohort (Sheardova et al., 2019) at the Memory Clinic of the Charles University, Second Faculty of Medicine, and Motol University Hospital in Prague, Czech Republic. All participants provided informed consent. The study was approved by the institutional ethics committee (no. EK – 701/16 25.5.2016). The participants with cognitive impairment were referred to the Memory Clinic by general practitioners and neurologists for memory complaints reported by participants themselves, their relatives, or health professionals. CN older adults were recruited from the University of the Third Age, senior centers (e.g., the Elpida center) or were relatives of participants and hospital staff (Parizkova et al., 2020).

All participants underwent clinical and laboratory evaluations, comprehensive cognitive assessment, brain MRI and the Navigation Test Suite. The participants with cognitive impairment underwent biomarker assessment including analysis of amyloid-β_1–42_, total tau, and p-tau_181_ in CSF or amyloid PET imaging or both, CSF biomarker assessment and amyloid PET imaging (Laczó et al., 2021b). They were classified as AD aMCI, non-AD aMCI, and mild AD dementia according to clinical diagnosis, and CSF biomarker and amyloid PET status. The participants with cognitive impairment were assigned to the relevant groups based on all available biomarkers, which had to be in agreement. All data were collected in 3–4 sessions within 60 days for each participant.

(i) Participants with AD aMCI (*n* = 33) met the clinical criteria for aMCI (Albert et al., 2011) including memory complaints, evidence of memory impairment [i.e., score lower than 1.5 standard deviations (SDs) below the age- and education-adjusted norms in any memory test], generally intact instrumental activities of daily living [<6 points on the Functional Activities Questionnaire, Czech Version (FAQ-CZ); Teng et al., 2010; Bezdíček et al., 2011] and absence of dementia. The participants had positive CSF AD biomarkers (reduced amyloid-β_1–42_ and elevated p-tau_181_ (<665 pg/ml and >48 pg/ml, respectively, the internally validated cut-offs; Parizkova et al., 2018; Laczó et al., 2021b; *n* = 9), positive amyloid PET imaging (positive visual read of 18F-flutemetamol PET scan; *n* = 16), or both, positive CSF AD biomarkers and amyloid PET imaging (*n* = 8).

(ii) Participants with non-AD aMCI (*n* = 31) met the clinical criteria for aMCI (Albert et al., 2011) and had negative amyloid-β biomarkers defined as normal CSF amyloid-β_1–42_ (≥665 pg/ml; *n* = 5), negative amyloid PET imaging (*n* = 19), or both, normal CSF amyloid-β_1–42_ and negative amyloid PET imaging (*n* = 7). A total of 60% of the participants with CSF biomarkers (*n* = 7) had elevated p-tau_181_ (>48 pg/ml; Parizkova et al., 2018; Laczó et al., 2021b) and about 60% of the participants with elevated p-tau_181_ (*n* = 4) also had elevated total tau (>358 pg/ml; Cerman et al., 2020).

(iii) Participants with mild AD dementia (*n* = 28) met the clinical criteria for dementia (Mckhann et al., 2011) with evidence of progressive cognitive impairment in at least two cognitive domains including memory (i.e., score lower than 1.5 SDs below the age- and education adjusted norms in any memory test and in at least one other non-memory cognitive test) and significant impairment in instrumental activities of daily living (≥6 points on the FAQ-CZ; Teng et al., 2010; Bezdíček et al., 2011). The participants had positive CSF AD biomarkers [reduced amyloid-β_1–42_ (<665 pg/ml) and elevated p-tau_181_ (>48 pg/ml; Parizkova et al., 2018; Laczó et al., 2021b) *n* = 14], positive amyloid PET imaging (*n* = 10), or both, positive CSF AD biomarkers and amyloid PET imaging (*n* = 4).

(iv) CN participants (*n* = 30) did not report any cognitive complaints and had cognitive performance within the normal range (i.e., score higher than 1.5 SDs below the age- and education-adjusted norms in all cognitive tests). In addition, they had no evidence of MTL atrophy on MRI. This was confirmed in all participants using the MTA visual scale on coronal T1-weighted (T1w) images, and the previously established age-specific MTA cut-off scores (i.e., score <2 in participants <75 years and score <3 in participants ≥75 years; Scheltens et al., 1992). Further, the participants did not have a family history of AD or other types of dementia in first-degree relatives. These criteria were applied to minimize the risk of including participants at increased risk of AD (i.e., individuals with subjective cognitive decline, hippocampal atrophy, or positive family history of AD).

#### Exclusion Criteria

Participants with depressive symptoms [≥6 points on the 15-item Geriatric Depression Scale (GDS-15)], anxiety [≥10 points on the Beck Anxiety Inventory (BAI)], low visual acuity [<20/40 (corrected) on visual acuity tests], moderate to severe white matter vascular lesions on MRI (Fazekas score >2 points), other primary neurological disorders (multiple sclerosis, epilepsy, Parkinsonian syndromes, and a history of traumatic brain injury or stroke) or psychiatric disorders (psychotic or schizoaffective disorders, major depressive disorder, anxiety disorders, and obsessive compulsive disorder), systemic diseases that can cause cognitive impairment, and a history of alcohol or drug abuse were not included in the study. In total, 131 participants from the CBAS cohort underwent spatial navigation assessment. The participants with cognitive impairment for whom biomarker data were not available at the time of the analysis were excluded (*n* = 4). Also, the participants with mild dementia for whom spatial navigation data were not available because they did not complete training in the Navigation Test Suite were excluded (*n* = 5).

### Spatial Navigation Assessment

**Figure 1:**
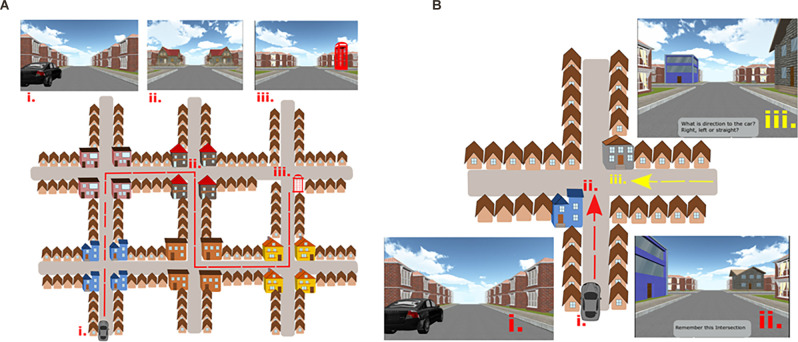
**(A)** The Navigation Test Suite with a schematic aerial view and corresponding screenshots from the Route-repetition and the Route-retracing tasks. Three points on the map are labeled: (i) The start location next to the car. (ii) One of the intersections along the route with gray houses at the corners of the intersection. (iii) The end of the route where the telephone box is present. In the Route-repetition task, the participants were passively transported through the city from the car to the telephone box during the encoding phase and in the test phase, the participants had to reproduce the same route. The Route-retracing task was identical to the Route-repetition task with the exception that participants in the test phase had to find their way back from the telephone box to the car. The order of intersections and houses at each intersection had a different design in each of these two tasks. **(B)** The Navigation Test Suite with a schematic aerial view and corresponding screenshots from the Directional-approach task: (i) Participants started the task next to the car. (ii) The encoding phase, where participants were passively transported toward one of the intersections featuring two unique houses. Participants had to remember where the car was parked. (iii) The test phase, where participants approached the intersection from a different direction (here from the east) and had to indicate the direction to the car. Figures 1A,B is adapted from Laczó et al. (2021a).

We used the Navigation Test Suite, which is described in detail in Wiener et al. (2020) and Laczó et al. (2021a). Here we reiterate the description of the test for better comprehension. The Navigation Test Suite consists of three navigation tasks: the Route-repetition task, the Route-retracing task, and the Directional-approach task. The Navigation Test Suite uses a virtual environment that consists of streets with residential houses and four-way intersections. The houses bordering the streets are all identical, except for the unique houses (i.e., distinct landmarks) that are located at each intersection (explained in detail below, Figures 1A,B). Participants could always see only one intersection at any time because the other more distant intersections were concealed in white fog. Prior to the testing, all participants completed familiarization training consisting of shorter versions of all three tasks (a three-intersection path for the Route-repetition and Route-retracing tasks and two separate intersections for the Directional-approach task; Wiener et al., 2020).

**(I) Route-Repetition Task**

In the encoding phase, the participants were positioned in a street next to a black car. They were then passively transported along a route featuring five intersections with one right turn, three left turns, and one straight movement. The route then stopped at a red phone box. Each intersection featured four identical houses at the four corners. Different intersections featured different houses (i.e., landmarks), such that each intersection had a unique appearance. Participants were instructed to remember the route (Figure 1A). In the test phase, the participants were asked to reproduce the same route from the car to the phone box. Participants were passively transported towards each of the intersections where they were stopped 20 m before the center of the intersections and were prompted to verbally indicate the direction in which the route continued. The examiner pressed a corresponding arrow key, and the participants were passively transported to the center of the intersection facing the street, which led to the following intersection. Thus, participants did not receive feedback. The task was composed of three experimental sessions along the same route to assess learning.

**(II) Route-Retracing Task**

The encoding phase was similar to the Route-repetition task. The route also comprised five intersections but featured different houses at intersections (Figure 1A). In the test phase, the participants had to navigate in the opposite direction compared to the encoding phase, i.e., from the endpoint of the route (the telephone box) back to the start (the black car). The Route-retracing task consisted of three identical consecutive sessions.

**(III) Directional-Approach Task**

The Directional-approach task assessed participants’ ability to encode the configuration of houses (landmarks) at an intersection and assessed perspective taking and wayfinding (Wiener et al., 2013; de Condappa and Wiener, 2016). The task consisted of 15 independent trials. Each trial began with an encoding phase, in which participants were positioned in a street next to a black car from where they were passively transported toward a single intersection, which featured two unique houses (i.e., landmarks) at diagonally opposite corners of the intersection. The movement stopped 20 m before the center of the intersections, such that both unique houses were in view. Two other houses at the corners of the intersection were identical to the other houses along the street. The participants’ task was to memorize, in which street the car was parked. Each of the 15 trials featured a different combination of unique houses at the corners of the intersections.

In the test phase, participants were passively transported toward the same intersection, but from one of the other streets. They were then asked to indicate the direction in which the car was parked (i.e., to indicate the street from which they originally approached the intersection). The movement stopped again 20 m before the center of the intersection, such that the unique houses could be seen.

The car was always parked in the street to the south of the intersection (Figure 1B). In the test phase, participants approached the intersection from the western, eastern, or northern street. The participants were not aware of these cardinal directions in the experiment, but the information about these cardinal directions was used in the analysis. Participants were required to perform perspective shifts to align the view during the test phase with that during the encoding phase. The perspective shift was 90° when approaching the intersection from the west or east and 180° when approaching from the north. In contrast to the Route-repetition and Route-retracing tasks, the Directional-approach task did not require participants to learn a route with multiple decision points.

### Cognitive Assessment

The cognitive assessment included the following tests: (1) verbal memory measured with the Rey Auditory Verbal Learning Test (RAVLT)—trials 1–5 and 30-min Delayed Recall trial (Bezdíček et al., 2014); (2) non-verbal memory measured with the Rey-Osterrieth Complex Figure Test (ROCFT)—the Recall condition after 3 min (Drozdova et al., 2015); (3) visuospatial function measured with the ROCFT—the Copy condition (Drozdova et al., 2015) and the Clock Drawing Test (Mazancova et al., 2017); (4) executive function measured with the Trail Making Test (TMT) B and Controlled Oral Word Association Test (Czech version with letters N, K, and P; Nikolai et al., 2018); (5) attention and working memory measured with the Forward and Backward Digit Spans and TMT A (Nikolai et al., 2018); and (6) language measured with the Boston Naming Test (30-item version) and Semantic Verbal Fluency test (Animals; Nikolai et al., 2018). The Mini-Mental State Examination (MMSE; Štěpánková et al., 2015) was administered to measure global cognitive function. The GDS-15 (Yesavage and Sheikh, 1986) and BAI (Beck et al., 1988) were used to assess depressive symptoms and anxiety in the participants. Group-wise neuropsychological characteristics are listed in Table 1.

**Table 1:** Characteristics of study participants.

	CN (*n* = 30)	Non-AD aMCI (*n* = 31)	AD aMCI (*n* = 33)	Mild AD dementia (*n* = 28)	*P* values	Effect sizes
*Demographic characteristics*
Age (years)	68.73 (5.82) **^c	70.32 (7.67)	72.27 (6.35)	74.32 (5.92)	<0.009	0.09
Women, n (%)	21 (70)	15 (48)	20 (61)	18 (64)	<0.361	0.16
Education (years)	16.20 (1.77)^**a^	13.87 (2.33)	14.79 (3.15)	14.43 (2.70)	<0.005	0.10
*Spatial navigation assessment*
Route-repetition task (% correct)	85.33 (13.33) ^***b-c^	74.41 (15.41) ^***b-c^	55.15 (19.31)	54.52 (17.41)	<0.001	0.34
Route-retracing task (% correct)	71.11 (19.60)^**a,***b-c^	54.22 (24.93)	40.81 (19.42)	40.00 (16.02)	<0.001	0.24
Directional-approach task (% correct)	71.56 (21.86) ^**a,***b-c^	54.00 (23.18)	44.65 (20.51)	37.62 (17.14)	<0.001	0.26
*Cognitive assessment*
MMSE	29.37 (0.89)^***a-c^	27.45 (2.31) ^***c^	26.27 (1.94)^***c^	22.11 (2.32)	<0.001	0.65
GDS-15 (score)	1.20 (2.02)^*a, **c^	3.00 (2.73)	2.25 (2.03)	3.32 (2.84)	<0.005	0.10
BAI (score)	5.40 (5.56)	7.58 (6.54)	8.82 (9.88)	7.43 (5.12)	<0.304	0.03
RAVLT 1–5 (score)	56.30 (7.72)^***a-c^	36.35 (9.57)^**c^	34.84 (8.20)^*c^	27.31 (4.77)	<0.001	0.64
RAVLT 30 (score)	12.07 (1.82)^***a-c^	5.03 (2.94)^*c^	3.92 (3.27)	2.25 (3.19)	<0.001	0.66
TMT A (seconds)	35.72 (10.94)^***c^	50.66 (23.41)^***c^	53.49 (29.06)^**c^	79.97 (41.82)	<0.001	0.24
TMT B (seconds)	79.58 (35.28)^***a-c^	160.53 (79.79)^***c^	161.40 (87.01) ^***c^	243.68 (75.69)	<0.001	0.39
COWAT (score)	50.87 (9.26) ^***a, c, b^	38.32 (11.40)	42.91 (10.58)^***c^	31.57 (13.16)	<0.001	0.28
ROCFT-C (score)	32.12 (2.18)^**a,***b-c^	27.55 (4.44) ^*c^	26.62 (5.55)	23.43 (8.17)	<0.001	0.24
ROCFT-R (score)	20.47 (5.29)^***a-c^	10.08 (5.32)^***c^	7.02 (5.63) ^*c^	2.87 (3.65)	<0.001	0.62
DSF (score)	10.07 (2.29)^**a, c^	8.35 (1.80)	9.03 (2.04)	8.07 (1.65)	<0.001	0.13
DSB (score)	6.80 (2.02)^*a, b,***c^	5.52 (1.63)	5.58 (1.80)	4.64 (1.22)	<0.001	0.17
CDT (score)	15.03 (1.19)^*b,***c^	14.42 (1.93)^***c^	13.72 (1.97)^***c^	11.75 (2.17)	<0.001	0.31
SVF Animals (score)	28.50 (4.47)^***a-c^	20.29 (5.78)^***c^	20.00 (4.22)^***c^	14.00 (4.01)	<0.001	0.55
BNT (no. of errors)	1.13 (1.31)^**a, b,***c^	4.39 (3.44)^**c^	4.39 (2.82) ^**c^	7.68 (4.91)	<0.001	0.32
*CSF analysis* ^d^
Amyloid-β_1_–42 (pg/ml)	N/A	999.80 (338.21) ^***b-c^	421.96 (83.89)	445.76 (128.79)	<0.001	0.63
Total tau (pg/ml)	N/A	303.66 (122.29)^**c^	607.13 (325.19)	824.92 (509.72)	<0.004	0.24
P-tau_181_ (pg/ml)	N/A	54.64 (21.06)^*c^	102.49 (72.18)	111.74 (60.32)	<0.033	0.15
*MRI brain measures* ^e^
Hippocampal head right^f^ (volume, cm^3^)	1.65 (0.24)^*c^	1.61 (0.25)	1.53 (0.30)	1.40 (0.33)	<0.015	0.10
Hippocampal head left^f^ (volume, cm^3^)	1.50 (0.24)*^c^	1.49 (0.26)	1.41 (0.28)	1.28 (0.28)	<0.016	0.10
Hippocampal body right^f^ (volume, cm^3^)	0.93 (0.11)^*b,***c^	0.94 (0.16)^*b,***c^	0.81 (0.14)	0.73 (0.14)	<0.001	0.27
Hippocampal body left^f^ (volume, cm^3^)	0.99 (0.13)^**b,***c^	0.93 (0.17) ^***c^	0.84 (0.16)	0.76 (0.12)	<0.001	0.27
Hippocampal tail right^f^ (volume, cm^3^)	0.28 (0.04) ^***b,***c^	0.27 (0.04) ^**b,***c^	0.23 (0.05) ^*c^	0.20 (0.04)	<0.001	0.40
Hippocampal tail left^f^ (volume, cm^3^)	0.30 (0.04) ^***b-c^	0.29 (0.05) ^**b,***c^	0.24 (0.06)	0.21 (0.04)	<0.001	0.36
alEC right^f^ (volume, cm^3^)	0.61 (0.10) ^*b,***c^	0.61 (0.10) ^*b,***c^	0.53 (0.08)	0.49 (0.10)	<0.001	0.24
alEC left^f^ (volume, cm^3^)	0.72 (0.10) ^***c^	0.70 (0.11) ^**c^	0.65 (0.11)	0.59 (0.10)	<0.001	0.18
pmEC right^f^ (volume, cm^3^)	0.34 (0.04) ^**b-c^	0.35 (0.04) ^**b-c^	0.30 (0.06)	0.29 (0.05)	<0.001	0.22
pmEC left^f^ (volume, cm^3^)	0.38 (0.04) ^***b-c^	0.37 (0.05) ^*b,***c^	0.32 (0.07)	0.30 (0.05)	<0.001	0.26
*MRI brain measures* ^e^
Caudate nucleus right^f^ (volume, cm^3^)	3.61 (0.45)	3.57 (0.46)	3.50 (0.58)	3.37 (0.50)	<0.342	0.04
Caudate nucleus left^f^ (volume, cm^3^)	3.60 (0.38)	3.50 (0.49)	3.45 (0.53)	3.37 (0.43)	<0.348	0.03
Precuneus right (thickness, mm)	2.26 (0.12) ^**b,***c^	2.19 (0.16) ^**c^	2.11 (0.16)	2.03 (0.18)	<0.001	0.23
Precuneus left (thickness, mm)	2.21 (0.14) ^***b-c^	2.18 (0.18) ^*b, **c^	2.02 (0.18)	1.99 (0.19)	<0.001	0.25
Isthmus cingulate right (thickness, mm)	2.26 (0.20) ^**b, *c^	2.18 (0.14)	2.07 (0.17)	2.10 (0.19)	<0.001	0.16
Isthmus cingulate left (thickness, mm)	2.32 (0.25) ^**b, *c^	2.24 (0.19)	2.07 (0.24)	2.12 (0.23)	<0.001	0.16
Posterior parietal cortex right (thickness, mm)	2.30 (0.13) ^***b-c^	2.26 (0.17) ^**b,***c^	2.10 (0.16)	2.06 (0.19)	<0.001	0.29
Posterior parietal cortex left (thickness, mm)	2.29 (0.12) ^***b-c^	2.24 (0.15) ^*b,***c^	2.10 (0.17)	2.04 (0.19)	<0.001	0.29

Demographic, cognitive, CSF, and MRI characteristics. Values are mean (SD) except for gender. *P* values refer to the main effect across all groups; *P* values indicate the level of significance; *p < 0.05; **p < 0.01; ***p < 0.001; Effect sizes were calculated as Cramér’s V for the χ^2^ test (gender) and partial eta-squared for one-way and mixed analyses of variance (all other variables). ^a^Differences compared to the non-AD aMCI group. ^b^Differences compared to the AD aMCI group. ^c^Differences compared to the mild AD dementia group. ^d^Based on a sample with CSF data (total *n* = 47) with non-AD aMCI (*n* = 12), AD aMCI (*n* = 17) and mild AD dementia (*n* = 18) participants. ^e^Based on a sample with complete brain imaging data (total *n* = 100) with CN (*n* = 29), non-AD aMCI (*n* = 23), AD aMCI (*n* = 26) and mild AD dementia (*n* = 22) participants. ^f^Volume normalized to estimated total intracranial volume. Key: CN, cognitively normal; non-AD aMCI, amnestic mild cognitive impairment with negative AD biomarkers; AD aMCI, amnestic mild cognitive impairment with Alzheimer’s disease; mild AD dementia, mild dementia with Alzheimer’s disease; MMSE, Mini-Mental State Examination; GDS-15, Geriatric Depression Scale 15-item version; BAI, Beck Anxiety Inventory; RAVLT, Rey Auditory Verbal Learning Test; RAVLT 1–5, trials 1–5 total; RAVLT 30, delayed word recall after 30 min; TMT A and B, Trail Making Tests A and B; COWAT, Controlled Oral Word Association Test (Czech version with letters N, K and P); ROCFT-C, Rey-Osterrieth Complex Figure Test—the Copy condition; ROCFT-R, Rey-Osterrieth Complex Figure Test—the Recall condition after 3 min; DSF, Digit Span Forward total score; DSB, Digit Span Backward total score; CDT, Clock Drawing Test—Cohen’s scoring; SVF, Semantic Verbal Fluency; BNT, Boston Naming Test (30-item version); CSF, cerebrospinal fluid; p-tau_181_, phosphorylated tau_181_; MRI, magnetic resonance imaging; alEC, anterolateral entorhinal cortex; pmEC, posteromedial entorhinal cortex.

### Magnetic Resonance Imaging

#### Image Acquisition

We used the established MRI protocol (Parizkova et al., 2018) performed on a Siemens Avanto 1.5T scanner (Siemens AG, Erlangen, Germany) with a 12-channel head coil. The 3-dimensional T1w (3D T1w) high-resolution magnetization-prepared rapid gradient echo (MPRAGE) sequence was used with the following parameters: TR/TE/TI = 2,000/3.08/1,100 ms, flip angle = 15°, 192 continuous partitions, slice thickness = 1.0 mm and in-plane resolution = 1 mm. Scans were visually inspected to ensure appropriate data quality and to exclude participants with a major brain pathology that could interfere with cognitive functioning. The 3D T1w images of high quality were available for 100 participants, including CN (*n* = 29), non-AD aMCI (*n* = 23), AD aMCI (*n* = 26), and mild AD dementia (*n* = 22). In the remaining participants (*n* = 22), the 3D T1w images were of low quality or unavailable. The demographic characteristics of participants with brain imaging data are presented in Supplementary Table 1.

#### Image Processing and Regional Brain Volumetry

We used a processing pipeline based on a population-based template and manual segmentation to measure volumes of the hippocampal head, body, and tail, volumes of the alEC and pmEC, and estimated total intracranial volume (eTIV). The processing pipeline is described in detail in Laczó et al. (2021b). In short, we created a population-based template, using structural 3D T1w images of 26 CN older adults recruited from CBAS (Sheardova et al., 2019). The 3D T1w images were processed using the freely available Advanced Normalization Tools package (ANTs^2^; Avants et al., 2011). An initial registration template was created first and we then proceeded to create the definite population template by registering 3D T1w images iteratively into the initial template. Manual segmentation of the hippocampus and EC was performed for each of 26 CN participants used for population-based template creation. The hippocampus and the EC were delineated manually in the coronal plane using anatomical landmarks according to the previously published manual segmentation protocol (Berron et al., 2017). The hippocampus was divided into three subregions—the head, the body, and the tail, and EC was divided into the alEC and pmEC subregions according to the previously published segmentation protocols (Berron et al., 2017; Olsen et al., 2017), respectively. The segmentation of the hippocampus and EC is described in detail in Laczó et al. (2021b). Manually delineated ROIs were normalized to MNI space using deformation fields obtained during the template creation. We created a template for each structure (i.e., the hippocampal head, body, and tail, alEC and pmEC) using the same procedure as during the initial template creation (Laczó et al., 2021b). Individual templates were split into the left and right masks using the left and right hemispheric ROIs. The resulting masks were rescaled into values 0–100 to represent probabilistic distribution.

The following steps were implemented to measure the subregional hippocampal and EC volumes of the study participants. We skull-stripped the individual 3D T1w images and performed B1 field intensity inhomogeneity correction using the N4 algorithm and performed three-tissue segmentation using statistical parametric mapping (SPM8, Wellcome Trust Center for Neuroimaging; Ashburner, 2009) and the VBM8-toolbox^3^ implemented in MatLab R2015b (MathWorks, Natick, MA). We then registered the CBAS template created in the previous step and diffeomorphically warped it into individual participants’ space using ANTs, with a cross-correlation method, 100 × 100 × 50 iterations, and symmetric normalization applied on a 0.25 threshold. The resulting warp field was used to transform ROI masks of individual hippocampal and EC subregions into the participants’ space. The ROIs masks were subsequently masked with a gray matter ROI and their volumes were extracted. Warps were visually inspected for accuracy, no volumes were removed. FreeSurfer 5.3 suite was used to calculate volumes of the right and left caudate nucleus and thickness of the right and left precuneus, isthmus cingulate, and composite region of the posterior parietal cortex. The processing details are described elsewhere (Dale et al., 1999; Fischl et al., 2002, 2004; Desikan et al., 2006) and are available online at http://surfer.nmr.mgh.harvard.edu/. Individual cortical and subcortical segmentations were projected onto corresponding skull-stripped brain images. Segmentations were visually assessed for accuracy and labeled as acceptable/non-acceptable in a binary fashion. No volumes were removed. Posterior parietal cortical thickness was computed as an area-weighted mean of superior parietal gyrus, inferior parietal gyrus, and supramarginal gyrus thickness obtained from FreeSurfer cortical parcellation using the Desikan-Killiany cortical atlas (Desikan et al., 2006). Volumes were normalized to eTIV using the previously published regression formula (Jack et al., 1992; Laczó et al., 2015).

### Cerebrospinal Fluid Analysis of AD Biomarkers

The CSF samples were obtained by lumbar puncture with an atraumatic needle in the lying position. The first 3 ml of CSF were used for routine analysis and the remaining 10 ml of CSF was centrifuged and stored at –80°C 30 min after the puncture. CSF collection, processing, and archiving were performed in accordance with European recommendations (Vanderstichele et al., 2012). CSF amyloid-β_1–42_, total tau, and p-tau_181_ were analyzed using ELISA (Innogenetics, Ghent, Belgium) in the Cerebrospinal Fluid Laboratory, Institute of Immunology and Department of Neurology, Second Faculty of Medicine, Charles University and Motol University Hospital. Unbiased cut-offs of less than 665 pg/ml and more than 48 pg/ml and 358 pg/ml were used to define amyloid-β_1–42_, p-tau_181_, and total tau positivity, respectively. These predefined cutoffs (Parizkova et al., 2018; Laczó et al., 2021b) were based on internal receiver operating characteristic (ROC) analyses and were validated against amyloid PET status in the Czech Brain Aging Study with 79% agreement and areas under the ROC curves (AUCs) of 85% (Cerman et al., 2020). The diagnosis of AD was made when both amyloid-β_1–42_ and p-tau_181_ were positive (Laczó et al., 2021b). CSF data were available for 47 of the 92 participants with cognitive impairment, including those with non-AD aMCI (*n* = 12), AD aMCI (*n* = 17), and mild AD dementia (*n* = 18). The demographic characteristics of participants with CSF data are presented in Supplementary Table 2.

### Amyloid PET Imaging

The PET images were acquired using a Biograph 40 TrueV HD PET/CT scanner (Siemens Healthineers AG, Erlangen, Germany) in the Department of Nuclear Medicine and PET Centre, Na Homolce Hospital. The participants received a single intravenous dose of flutemetamol (18F; Vizamyl, GE Healthcare, Chicago, IL) with a gross activity of 206.7 ± 12.7 MBq. Non-contrast low-dose CT brain images were acquired for attenuation correction prior to the PET scans. A PET list-mode acquisition was performed in two phases. The early-phase images were acquired at the time of flutemetamol (18F) administration for 8 min and rebinned into dynamic datasets of 2 × 4 min for motion checking. They were iteratively reconstructed to a 168 × 168 matrices with three iterations, after attenuation, scatter, and point spread function correction. The late-phase images were acquired 90 min after flutemetamol (18F) administration for 10 min and iteratively reconstructed to a 128 × 128 matrix with other parameters as described above, including rebinning into dynamic sequences for motion checking (Belohlavek et al., 2019). Flutemetamol (18F) PET images were visually read (as positive or negative) by two independent physicians certified in nuclear medicine, who were previously in-person trained and qualified. The late-phase images were evaluated for amyloid-β-specific uptake in the gray matter by the method that visualized the gray-white matter borders derived from the early-phase images (GM-EDGE method; Belohlavek et al., 2019). Eight specific brain regions were assessed, including the frontal lobe, lateral temporal lobe, anterior cingulate, posterior cingulate, precuneus, temporoparietal area, insula, and striatum. If any of these regions was abnormal, the finding was classified as positive for amyloid-β. The GM-EDGE method was shown to have a high inter-rater agreement (> 90%; Belohlavek et al., 2019) and good concordance with amyloid-β_1–42_ levels in CSF (≈80%; Cerman et al., 2020). Amyloid PET imaging data were available for 64 of the 92 participants with cognitive impairment, including those with non-AD aMCI (*n* = 26), AD aMCI (*n* = 24), and mild AD dementia (*n* = 14).

### Statistical Analysis

A one-way analysis of variance (ANOVA) with *post-hoc* Sidak’s test evaluated differences between the groups in continuous demographic variables, cognitive performance, CSF biomarkers, and MRI brain measures. A χ^2^ test evaluated differences in gender proportions. A mixed-model analysis of covariance (ANCOVA) with the diagnostic group (CN vs. non-AD aMCI vs. AD aMCI vs. mild AD dementia) as the between-subjects factor and the session (1st vs. 2nd vs. 3rd) or the approach direction (east vs. north vs. west) as the within-subjects factors were used to analyze differences in spatial navigation performance measured as the percentage of correct responses, which was the dependent variable. The analysis was controlled for age (mean-centered), years of education (mean-centered), and gender. The intercept, session/approach direction, and a person identifier were specified as random effects. Based on model fit, the final models used the scaled identity covariance structure. The *post-hoc* Sidak’s test was used to assess differences between the individual groups and sessions/approach directions. The *post-hoc* pairwise comparisons with Holm–Bonferroni (H-B) correction for multiple comparisons were used to compare differences in spatial navigation performance between individual groups in each session/approach direction and to interpret significant interactions between variables. A one-sample t-test was used to assess differences from chance performance (i.e., 33.33%) for each diagnostic group in each session/approach direction. The *post-hoc* ROC analysis was used to assess the ability of the spatial navigation tasks to differentiate the CN, non-AD aMCI, AD aMCI, and mild AD dementia groups. Sizes of the AUCs, sensitivity, specificity, and optimal cut-off values based on the Youden’s index were calculated.

**Figure 2:**
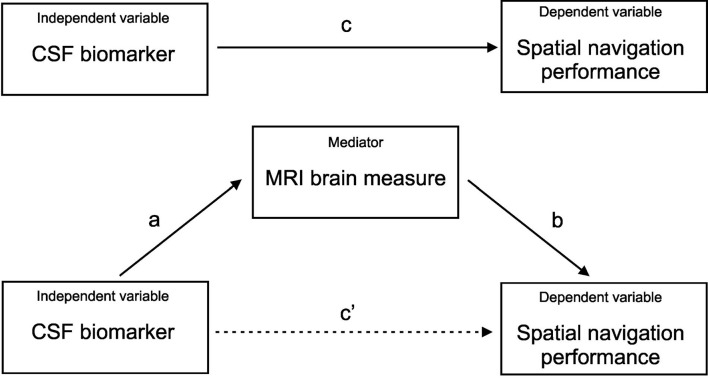
Mediation analysis. CSF AD biomarkers (the independent variable); spatial navigation performance in each task (the dependent variable); MRI brain measures (the mediator); c) total effect of the independent variable on the dependent variable; c’) direct effect of the independent variable on the dependent variable controlled for the mediator; a*b) indirect effect of the independent variable on the dependent variable through the mediator.

Pearson’s correlation coefficients were calculated to explore the associations between MRI measures of atrophy in nine specific brain regions for the right and left hemispheres, and performance in three Navigation Test Suite tasks. The H-B correction for multiple comparisons across the 54 pairings was used. Next, the separate linear regression models adjusted for age (mean-centered), years of education (mean-centered), and gender were used to control for the effect of demographic characteristics on the significant associations. To address the specific hypothesis that each CSF biomarker (i.e., amyloid-β_1–42_, total tau, and p-tau_181_) will be differently associated with performance in each of the Navigation Test Suite tasks (i.e., the Route-repetition, Route-retracing, and Directional-approach tasks), we calculated Pearson’s correlation coefficients and if the associations were significant (uncorrected), we used the linear regression model adjusted for age (mean-centered), years of education (mean-centered) and gender. Mediation (path) analyses were conducted to assess the associations between CSF AD biomarkers (the independent variable) and spatial navigation performance in each task (the dependent variable) with MRI brain measures serving as the mediator. Only variables significantly associated with spatial navigation performance in the previous regression analyses were included in the mediation analyses. The analyses were adjusted for age (mean-centered), education (mean-centered), and gender. The bootstrapping method (Hayes, 2013) was used to test for the significance of the indirect effect with a 95% confidence interval (CI). Variable values were converted to z-scores prior to the mediation analyses to present the mediation effects and 95% CI in standardized units. In the mediation analysis (Figure 2), the “c path” (total effect; TE) represents the effect of the independent variable on the dependent variable, the “c’ path” (direct effect; DE) represents the effect of the independent variable on the dependent variable while accounting for the mediator, the “a path” represents the relationship between the independent variable and the mediator, and the “b path” represents the relationship between the mediator and the dependent variable. The last two paths form the “a*b path” (indirect effect; IE) that represents the effect of the independent variable on the dependent variable through the mediator.

Statistical significance was set at two-tailed (alpha) of 0.05. Effect sizes are reported using partial eta-squared ($\eta_{p}^{2}$) for one-way ANOVA and mixed-model ANCOVA. A partial eta-squared of 0.2 corresponds to Cohen’s d of 1.0. All analyses were conducted using IBM SPSS 27.0 software.

## Results

### Demographics and Cognitive Performance

The demographic characteristics are presented in detail in Table 1. The CN group was younger than the mild AD dementia group (*p* = 0.008) and more educated than the non-AD aMCI group (*p* = 0.003). There were no significant differences in gender between the groups. As expected, the non-AD aMCI, AD aMCI, and mild AD dementia groups had lower MMSE scores (all *p* ≤ 0.001) and worse cognitive performance in most of the tests (*p* ≤ 0.036) compared to the CN group. The non-AD aMCI and AD aMCI groups did not differ in cognitive performance (all *p* ≥ 0.100). The non-AD aMCI (*p* = 0.028) and mild AD dementia (*p* = 0.007) groups reported more depressive symptoms than the CN group. There were no significant differences in anxiety symptoms between the groups.

### Spatial Navigation Performance

**Figure 3:**
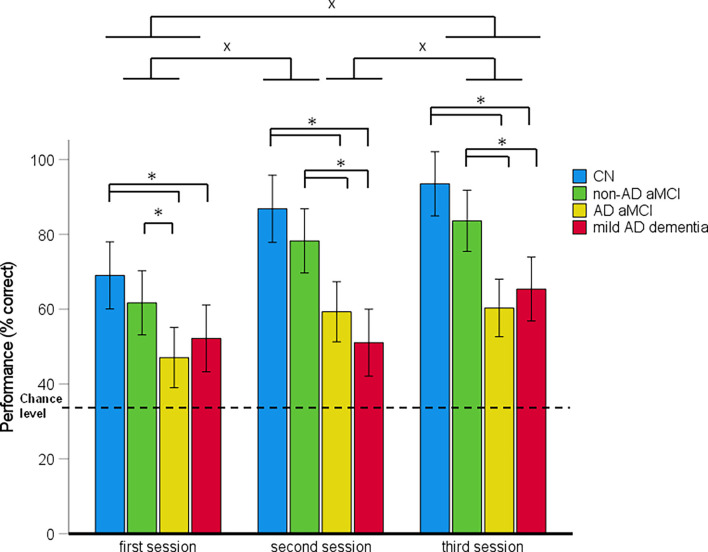
Route-repetition task—spatial navigation performance as mean percentage of correct responses in each session (95% Cl). *p < 0.05 indicating the differences between the groups; ^x^*p* < 0.05 indicating the differences between the sessions. CN, cognitively normal; non-AD aMCI, amnestic mild cognitive impairment with negative AD biomarkers; AD aMCI, amnestic mild cognitive impairment with Alzheimer’s disease; mild AD dementia, mild dementia with Alzheimer’s disease; CI, confidence interval.

**Figure 4:**
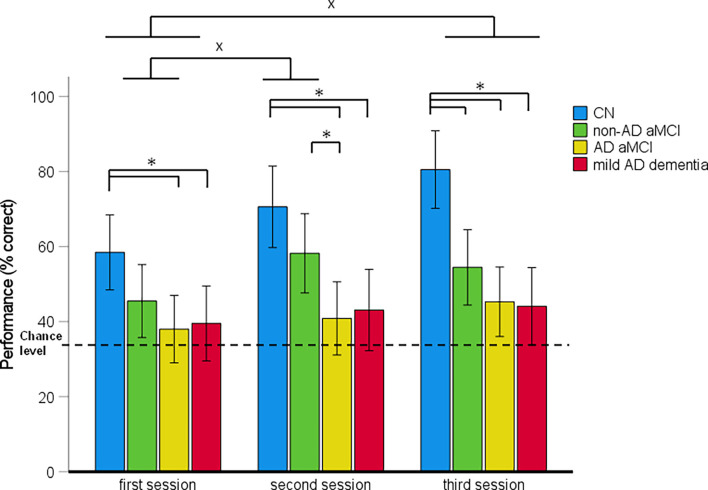
Route-retracing task—spatial navigation performance as mean percentage of correct responses in each session (95% Cl). *p < 0.05 indicating the differences between the groups; ^x^*p* < 0.05 indicating the differences between the sessions. CN, cognitively normal; non-AD aMCI, amnestic mild cognitive impairment with negative AD biomarkers; AD aMCI, amnestic mild cognitive impairment with Alzheimer’s disease; mild AD dementia, mild dementia with Alzheimer’s disease; CI, confidence interval.

**Figure 5:**
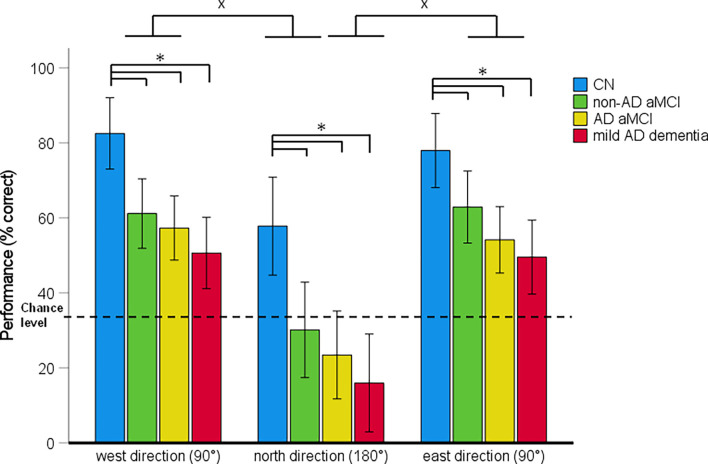
Directional-approach task—spatial navigation performance as mean percentage of correct responses in each approach direction (95% Cl). *p < 0.05 indicating the differences between the groups; ^x^*p* < 0.05 indicating the differences between the approach directions. CN, cognitively normal; non-AD aMCI, amnestic mild cognitive impairment with negative AD biomarkers; AD aMCI, amnestic mild cognitive impairment with Alzheimer’s disease; mild AD dementia, mild dementia with Alzheimer’s disease; CI, confidence interval.

Spatial navigation performance shown as the mean percentage of correct responses in the CN, non-AD aMCI, AD aMCI, and mild AD dementia groups for each Navigation Test Suite task is presented in Figures 3–5.

#### Route-Repetition Task Performance

In the 4 (diagnostic group) × 3 (session) mixed-model ANCOVA, there was a significant main effect of diagnostic group (*F*_(3,122)_ = 20.67, *p* < 0.001, $\eta_{p}^{2}$ = 0.34) and session (*F*_(2,244)_ = 26.96, *p* < 0.001, $\eta_{p}^{2}$ = 0.19; Figure 3). The session-by-diagnostic group interaction was not significant (*F*_[6,244]_ = 1.98, *p* = 0.069, $\eta_{p}^{2}$ = 0.04). On average, the AD aMCI group performed worse than the non-AD aMCI group [*p* < 0.001, 95% CI (−29.76, −8.17)] and the CN group [*p* < 0.001, 95% CI (−38.69, −16.44)], and did not differ from the mild AD dementia group [*p* = 1.00, 95% CI (−11.58, 10.27)]. The non-AD aMCI group did not differ from the CN group [*p* = 0.272, 95% CI (−20.29, 3.09)] and performed better than the mild AD dementia group [*p* < 0.001, 95% CI (6.92, 29.71)]. The mild AD dementia group performed worse than the CN group [*p* < 0.001, 95% CI (−38.83, −14.99)]. On average, spatial navigation performance improved across all sessions. Performance in the second session was better than that in the first session [*p* < 0.001, 95% CI (5.35, 17.40)] and performance in the third session was better than that in the first [*p* < 0.001, 95% CI (12.19, 24.24)] and second [*p* = 0.020, 95% CI (0.81, 12.86)] sessions.

The analysis of between-group differences in each session showed that the AD aMCI group performed worse than the non-AD aMCI (all p_H-Bcorrected_ ≤ 0.031) and CN (all p_H-Bcorrected_ ≤ 0.002) groups in all three sessions. The mild AD dementia group performed worse than the CN group in all three sessions (all p_H-Bcorrected_ < 0.050) and worse than the non-AD aMCI group in the second and the third session (both p_H-Bcorrected_ ≤ 0.011). Other differences between the diagnostic groups were not significant. All groups performed above the chance level in all sessions [CN group: (all *t*_(29)_ ≥ 8.60, *p* < 0.001); non-AD aMCI group: (all *t*_(30)_ ≥ 7.10, *p* < 0.001); AD aMCI group: (all *t*_(32)_ ≥ 3.02, *p* ≤ 0.005); mild AD dementia group: (all *t*_(27)_ ≥ 3.13, *p* ≤ 0.004)].

In the *post-hoc* ROC analyses, performance in the Route-repetition task differentiated the CN group from the non-AD aMCI, AD aMCI and mild AD dementia groups with AUC values of 0.719 [95% CI (0.59, 0.85), *p* = 0.001], 0.89 [95% CI (0.81, 0.98), *p* < 0.001], and 0.92 [95% CI (0.85, 0.99), *p* < 0.001], respectively, the non-AD aMCI group from the AD aMCI and mild AD dementia groups with AUC values of 0.78 [95% CI (0.66, 0.89), *p* < 0.001] and 0.80 [95% CI (0.69, 0.91), *p* < 0.001], respectively, and did not differentiate the AD aMCI group from the mild AD dementia group, where the AUC value was 0.63 [95% CI (0.49, 0.77), *p* = 0.083]. The sensitivity and specificity for the relevant cut-off values is listed in Supplementary Table 3.

#### Route-Retracing Task Performance

In the 4 (diagnostic group) × 3 (session) mixed-model ANCOVA, there was a significant main effect of diagnostic group (*F*_[3,121]_ = 12.83, *p* < 0.001, $\eta_{p}^{2}$ = 0.24) and session (*F*_[2,242]_ = 7.47, *p* < 0.001, $\eta_{p}^{2}$ = 0.06; Figure 4). The session-by-diagnostic group interaction was not significant (*F*_[6,242]_ = 1.16, *p* = 0.331, $\eta_{p}^{2}$ = 0.03). On average, the AD aMCI group performed worse than the CN group [*p* < 0.001, 95% CI (−41.88, −15.07)] and did not differ from the non-AD aMCI [*p* = 0.128, 95% CI (−24.48, 1.80)] and mild AD dementia [*p* = 1.00, 95% CI (−14.02, 12.31)] groups. The non-AD aMCI group performed worse than the CN group [*p* = 0.009, 95% CI (−31.30, −2.96)] and did not differ from the mild AD dementia group [*p* = 0.244, 95% CI (−3.40, 24.37)]. The mild AD dementia group performed worse than the CN group [*p* < 0.001, 95% CI (−41.99, −13.25)]. On average, spatial navigation performance improved across the sessions. Performance in the second session was better than that in the first session [*p* = 0.021, 95% CI (0.91, 14.71)]. Performance in the third session was better than that in the first session (*p* < 0.001, 95% CI (3.83, 17.62)] but did not differ from that in the second session [*p* = 0.672, 95% CI (−3.98, 9.81)].

The analysis of between-group differences in each session showed that the AD aMCI group performed worse than the non-AD aMCI group in the second session (p_H-Bcorrected_ = 0.032) and worse than the CN group in all three sessions (all p_H-Bcorrected_ ≤ 0.016). The non-AD aMCI group performed worse than the CN group in the third session (p_H-Bcorrected_ = 0.003). The mild AD dementia group performed worse than the CN group in all three sessions (all p_H-Bcorrected_ < 0.050). Other differences between the diagnostic groups were not significant. The CN and non-AD aMCI groups performed above the chance level in each session [CN group: (all *t*_(29)_ ≥ 5.16, *p* < 0.001); non-AD aMCI group: (all *t*_(30)_ ≥ 2.81, *p* ≤ 0.009)]. The AD aMCI group performed above the chance level in the third session (*t*_(32)_ = 2.34, *p* = 0.026) and did not differ from the chance level in the first and the second session (both *t*_(27)_ ≤ 1.24, *p* ≥ 0.223). The mild AD dementia group did not differ from the chance level in any session (all *t*_(27)_ ≤ 1.85, *p* ≥ 0.075).

In the *post-hoc* ROC analyses, performance in the Route-retracing task differentiated the CN group from the non-AD aMCI, AD aMCI and mild AD dementia groups with AUC values of 0.68 [95% CI (0.56, 0.83), *p* = 0.004], 0.86 [95% CI (0.77, 0.95), *p* < 0.001], and 0.89 [95% CI (0.81, 0.97), *p* < 0.001], respectively, the non-AD aMCI group from the AD aMCI and mild AD dementia groups with AUC values of 0.64 [95% CI (0.51, 0.78), *p* = 0.041] and 0.65 [95% CI (0.51, 0.80), *p* = 0.034], respectively, and did not differentiate the AD aMCI group from the mild AD dementia group, where the AUC value was 0.50 [95% CI (0.35, 0.65), *p* = 0.075]. The sensitivity and specificity for the relevant cut-off values is listed in Supplementary Table 3.

#### Directional-Approach Task Performance

In the 4 (diagnostic group) × 3 (approach direction) mixed-model ANCOVA, there was a significant main effect of diagnostic group (*F*_[3,121]_ = 14.16, *p* < 0.001, $\eta_{p}^{2}$ = 0.26) and approach direction (*F*_[2,242]_ = 64.19, *p* < 0.001, $\eta_{p}^{2}$ = 0.36; Figure 5). The approach direction-by-diagnostic group interaction was not significant (*F*_[6,242]_ = 0.26, *p* = 0.955, $\eta_{p}^{2}$ = 0.01). On average, the non-AD aMCI [*p* = 0.001, 95% CI (−36.14, −6.59)], AD aMCI [*p* < 0.001, 95% CI (−41.78, −13.82)] and mild AD dementia [*p* < 0.001, 95% CI (−49.03, −19.06)] groups performed worse than the CN group. Other differences between the diagnostic groups were not significant. On average, spatial navigation performance varied depending on the approach direction. Performance was worse for an approach from the north (180° shift) than for an approach from the west [90° shift; *p* < 0.001, 95% CI (−38.46, −23.65)] and the east [90° shift; *p* < 0.001, 95% CI (−36.69, −21.88)]. Performance for the west and east approaches did not differ from each other [*p* = 0.918, 95% CI (−5.63, 9.17)].

The analysis of between-group differences for each approach direction showed that the non-AD aMCI (all p_H-Bcorrected_ ≤ 0.044), AD aMCI (all p_H-Bcorrected_ ≤ 0.002), and mild AD dementia (all p_H-Bcorrected_ < 0.001) groups performed worse than the CN group for all three approach directions. Other differences between the diagnostic groups were not significant. The CN group performed above the chance level for all approach directions (all *t*_(29)_ ≥ 2.71, *p* ≤ 0.011). The non-AD aMCI, AD aMCI and mild AD dementia groups performed above the chance level for an approach from the west and the east (non-AD aMCI group: [both *t*_(30)_ ≥ 5.70, *p* < 0.001]; AD aMCI group: (both *t*_(32)_ ≥ 4.60, *p* < 0.001); mild AD dementia group: (both *t*_(27)_ ≥ 3.27, *p* ≤ 0.003). For an approach from the north, performance in the non-AD aMCI (*t*_(30)_ = 0.31, *p* = 0.762) and AD aMCI (*t*_(32)_ = −1.71, *p* = 0.098) groups did not differ from the chance level and the mild AD dementia group performed below the chance level (*t*_(27)_ = −4.13, *p* < 0.001).

In the *post-hoc* ROC analysis, performance in the Directional-approach task differentiated the CN group from the non-AD aMCI, AD aMCI, and mild AD dementia groups with AUC values of 0.717 (95% CI [0.59, 0.85], *p* = 0.001), 0.81 (95% CI [0.69, 0.92], *p* < 0.001), and 0.88 (95% CI [0.79, 0.97], *p* < 0.001), respectively, the non-AD aMCI group from the mild AD dementia group with AUC value of 0.71 (95% CI [0.58, 0.84], *p* = 0.002), and did not differentiate the non-AD aMCI group from the AD aMCI group and the AD aMCI group from the mild AD dementia group, where the AUC values were 0.62 (95% CI [0.47, 0.76], *p* = 0.109), and 0.60 (95% CI [0.46, 0.74], *p* = 0.166), respectively. The sensitivity and specificity for the relevant cut-off values is listed in Supplementary Table 3.

### Association Between Regional Brain Atrophy and Spatial Navigation Performance

The MRI brain measures are summarized in Table 1. The AD aMCI group had lower volumes of the right and the left hippocampal body, tail and pmEC, the right alEC, and lower thickness of the right and left isthmus cingulate/RSC, precuneus, and posterior parietal cortex than the CN group (all *p* ≤ 0.015). The same was true for the mild AD dementia group, which in addition had lower volumes of the right and left hippocampal head and the left alEC than the CN group (all *p* ≤ 0.024). The AD aMCI group had lower volumes of the right and the left hippocampal tail, the right hippocampal body, and alEC, the right and left pmEC, and lower thickness of the left precuneus and the right and left posterior parietal cortex than the non-AD aMCI (all *p* ≤ 0.020). There were no significant differences in regional brain atrophy between the non-AD aMCI and the CN group (all *p* ≥ 0.533).

In the correlational analysis with HB correction (Table 2, Supplementary Figure 1), volume of the right alEC and thickness of the right and left precuneus and posterior parietal cortex were correlated with spatial navigation performance in the Route-repetition task (all *r* ≥ 0.38, *p* ≤ 0.001), volumes of the right hippocampal body and the right and left pmEC were correlated with spatial navigation performance in the Route-retracing task (all *r* ≥ 0.34, *p* ≤ 0.001), and volumes of the left hippocampal body, the right hippocampal tail and alEC, the right and left pmEC, and thickness of the right isthmus cingulate/RSC, the right and left precuneus and posterior parietal cortex were correlated with spatial navigation performance in the Directional-approach task (all *r* ≥ 0.32, *p* ≤ 0.001). All the associations but the association between volume of the right alEC and performance in the Directional-approach task remained significant in the regression analyses adjusted for age, education, and gender, where lower volumes and thickness were associated with worse spatial navigation performance (all ß ≥ 0.24, *p* ≤ 0.030; Table 3).

**Table 2:** Correlation matrix of spatial navigation performance and MRI brain measures.

	Route-repetition task (% correct)	Route-retracing task (% correct)	Directional-approach task (% correct)
Hippocampal head right^a^ (volume, cm^3^)	0.279**	0.237*	0.160
Hippocampal head left^a^ (volume, cm^3^)	0.226*	0.232*	0.132
Hippocampal body right^a^ (volume, cm^3^)	0.314**	0.345***	0.299**
Hippocampal body left^a^ (volume, cm^3^)	0.261**	0.293**	0.0.397***
Hippocampal tail right^a^ (volume, cm^3^)	0.303**	0.300**	0.0.392***
Hippocampal tail left^a^ (volume, cm^3^)	0.220*	0.239*	0.300**
alEC right^a^ (volume, cm^3^)	0.374***	0.288**	0.323**
alEC left^a^ (volume, cm^3^)	0.250*	0.265**	0.256**
pmEC right^a^ (volume, cm^3^)	0.301**	0.344***	0.0.345***
pmEC left^a^ (volume, cm^3^)	0.277**	0.340***	0.0.379***
Caudate nucleus right^a^ (volume, cm^3^)	0.156	0.226*	0.208*
Caudate nucleus left^a^ (volume, cm^3^)	0.118	0.195	0.164
Precuneus right (thickness, mm)	0.380***	0.262**	0.0.361***
Precuneus left (thickness, mm)	0.446***	0.286**	0.0.359***
Isthmus cingulate right (thickness, mm)	0.283**	0.249*	00.326**
Isthmus cingulate left (thickness, mm)	0.249*	0.167	0.200*
Posterior parietal cortex right (thickness, mm)	0.444***	0.254*	0.0.339***
Posterior parietal cortex left (thickness, mm)	0.419***	0.254*	0.0.342***

*Correlation (uncorrected) is significant at the 0.05 level (2-tailed), **Correlation (uncorrected) is significant at the 0.01 level (2-tailed), ***Correlation (uncorrected) is significant at the 0.001 level (2-tailed). Values in bold are significant after Holm-Bonferroni correction for multiple comparisons. ^a^Volume normalized to estimated total intracranial volume. Key: MRI, magnetic resonance imaging; alEC, anterolateral entorhinal cortex; pmEC, posteromedial entorhinal cortex.

**Table 3:** Regression analyses of spatial navigation performance and MRI brain measures controlled for demographic characteristics.

MRI brain measures	Spatial navigation performance (% correct)			
	Standardized Regression Coefficient β	Unstandardized Regression Coefficient B	Standard Error of Measurement SE	R Square
	*Route-repetition task*			
alEC right^a^ (volume, cm^3^)	0.276*	0.053*	0.021	0.224
Precuneus right (thickness, mm)	0.278**	33.077**	11.147	0.272
Precuneus left (thickness, mm)	0.360***	38.799***	9.959	0.319
Posterior parietal cortex right (thickness, mm)	0.346***	38.666***	10.675	0.306
Posterior parietal cortex left (thickness, mm)	0.299**	33.528**	11.034	0.279
	*Route-retracing task*			
Hippocampal body right^a^ (volume, cm^3^)	0.243*	0.037*	0.016	0.173
pmEC right^a^ (volume, cm^3^)	0.239*	0.106*	0.049	0.162
pmEC left^a^ (volume, cm^3^)	0.246*	0.094*	0.039	0.170
	*Directional-approach task*			
Hippocampal body left^a^ (volume, cm^3^)	0.319**	0.046**	0.014	0.197
Hippocampal tail right^a^ (volume, cm^3^)	0.316**	0.146**	0.046	0.197
pmEC left^a^ (volume, cm^3^)	0.311**	0.120**	0.039	0.175
pmEC right^a^ (volume, cm^3^)	0.261*	0.118*	0.050	0.144
alEC right^a^ (volume, cm^3^)	0.225	0.050	0.026	0.128
Precuneus left (thickness, mm)	0.340***	44.416***	12.740	0.245
Precuneus right (thickness, mm)	0.327***	47.143***	13.820	0.238
Posterior parietal cortex left (thickness, mm)	0.304**	40.903**	13.807	0.220
Posterior parietal cortex right (thickness, mm)	0.308**	41.174**	13.541	0.223
Isthmus cingulate right (thickness, mm)	0.258**	33.406**	12.535	0.203

*p < 0.05; **p < 0.01; and ***p < 0.001. ^a^Volume normalized to estimated total intracranial volume. Key: MRI, magnetic resonance imaging; alEC, anterolateral entorhinal cortex; pmEC, posteromedial entorhinal cortex.

### Association Between CSF Biomarkers and Spatial Navigation Performance

The CSF biomarker characteristics are presented in detail in Table 1. As expected, the non-AD aMCI group had higher CSF levels of amyloid-β_1–42_ than the AD aMCI and mild AD dementia groups (both *p* < 0.001). There were no significant differences in CSF levels of amyloid-β_1–42_ between the AD aMCI and the mild AD dementia group (*p* = 0.978). The non-AD aMCI group had lower CSF levels of total tau (*p* = 0.003) and p-tau_181_ (*p* = 0.038) than the mild AD dementia group. Other differences in CSF levels of total tau and p-tau_181_ between the diagnostic groups were not significant. There was a strong correlation between CSF levels of total tau and p-tau_181_ (*r* = 0.62, *p* < 0.001). CSF levels of p-tau_181_ and total tau negatively correlated with CSF levels of amyloid-β_1–42_ (both *r* = −0.29, *p* < 0.05).

In the correlational analysis with spatial navigation tasks (Table 4, Supplementary Figure 2), CSF levels of amyloid-β_1–42_ correlated with performance in the Route-repetition and Directional-approach tasks (both *r* ≥ 0.31, *p* ≤ 0.032), CSF levels of total tau correlated with performance in the Directional-approach task (*r* = −0.31, *p* = 0.041) and CSF levels of p-tau_181_ correlated with performance in the Route-retracing and Directional-approach tasks (both *r* ≥ −0.30, *p* ≤ 0.043). In the regression analyses adjusted for age, education and gender (Table 5), lower levels of amyloid-β_1–42_ were associated with worse spatial navigation performance in the Route-repetition task (*ß* = 0.39, *p* = 0.005) and higher levels of p-tau_181_ were associated with worse spatial navigation performance in the Route-retracing (*ß* = −0.28, *p* = 0.041) and Directional-approach (*ß* = −0.29, *p* = 0.037) tasks. Other associations in the regression analyses were not significant.

**Table 4:** Correlation matrix of spatial navigation performance and CSF biomarkers.

	Route-repetition task (% correct)	Route-retracing task (% correct)	Directional-approach task (% correct)
Amyloid-β_1–42_ (pg/ml)	0.425**	0.234	0.310*
Total tau (pg/ml)	−0.212	−0.290	−0.307*
P-tau_181_ (pg/ml)	−0.203	−0.296*	−0.329*

*Correlation (uncorrected) is significant at the 0.05 level (2-tailed), **Correlation (uncorrected) is significant at the 0.01 level (2-tailed). Key: CSF, cerebrospinal fluid; p-tau_181_, phosphorylated tau_181_.

**Table 5:** Regression analysis of spatial navigation performance and CSF biomarkers controlled for demographic characteristics.

CSF biomarkers	Spatial navigation performance (% correct)			
	Standardized Regression Coefficient β	Unstandardized Regression Coefficient B	Standard Error of Measurement SE	R Square
	*Route-repetition task*			
Amyloid-β_1–42_ (pg/ml)	0.394**	0.024**	0.008	0.289
	*Route-retracing task*			
P-tau_181_ (pg/ml)	-0.283*	-0.100*	0.048	0.265
	*Directional-approach task*			
Amyloid-β_1–42_ (pg/ml)	0.241	0.016	0.009	0.243
Total tau (pg/ml)	-0.215	-0.011	0.007	0.232
P-tau_181_ (pg/ml)	-0.298*	-0.098*	0.045	0.270

*p < 0.05; **p < 0.01. Key: CSF, cerebrospinal fluid; p-tau_181_, phosphorylated tau_181_.

In the mediation analyses adjusted for age, education, and gender, in the Route-repetition task, lower levels of amyloid-β_1–42_ were directly associated with worse spatial navigation performance when using the thickness of the right and left precuneus and posterior parietal cortex as the mediators [all DE ≥ 0.28, 95% CI (0.00, 0.55) to (0.02, 0.58), all *p* ≤ 0.049]. The direct effect was not significant when using volume of the right alEC as the mediator [DE = 0.24, 95% CI (−0.07, 0.55), *p* = 0.119]. The indirect effect was not significant in any of the analyses with the Route-repetition task performance [all IE ≤ 0.08, 95% CI (−0.12, 0.34) to (−0.04, 0.11)]. In the Route-retracing task, higher levels of p-tau_181_ were directly associated with worse spatial navigation performance when using volumes of the right and left pmEC as the mediators [both DE ≥ −0.35, 95% CI (−0.65, −0.05) to (−0.66, −0.07), both *p* ≤ 0.023]. The direct effect was not significant when using volume of the right hippocampal body as the mediator [DE = −0.30, 95% CI (−0.61, 0.00), *p* = 0.052]. The indirect effect was not significant in any of the analyses with the Route-retracing task performance [all IE ≤ −0.05, 95% CI (−0.06, 0.05) to (−0.19, 0.01)]. In the Directional-approach task, higher levels of p-tau_181_ were not directly associated with spatial navigation performance when using the thickness of the right and left precuneus and posterior parietal cortex, the thickness of the right isthmus cingulate/RSC and volumes of the right pmEC and hippocampal tail and the left hippocampal body as the mediators [all DE ≤ −0.27, 95% CI (−0.50, 0.09) to (−0.58, 0.03), all *p* ≥ 0.074]. The direct effect was significant when using volume of the left pmEC as the mediator [DE = −0.29, 95% CI (−0.58, −0.00), *p* = 0.047]. However, the total effect was not significant in this mediation analysis (TE = −0.28 95% CI [−0.57, 0.22], *p* = 0.068), therefore, the significant direct effect is difficult to interpret. The indirect effect was not significant in any of the analyses with the Directional-approach task performance [all IE ≤ −0.07, 95% CI (−0.04, 0.05) to (−0.36, 0.03)]. The results of the mediation analyses are described in detail in Supplementary Table 4. Correlations between spatial navigation performance and MRI brain measures in the subsample used for the mediation analyses, and correlations between CSF biomarkers and MRI brain measures are described in Supplementary Tables 5, 6.

## Discussion

We examined the differences in spatial navigation performance between the AD biomarker positive and negative aMCI participants in various tasks of the navigation test using a realistic-looking virtual environment. Next, we explored the relationships between spatial navigation performance and MRI measures of atrophy in the specific MTL, cortical and subcortical regions. Finally, we examined the associations of performance with CSF AD biomarkers and the role of regional brain atrophy in these associations. We found that the AD aMCI participants performed generally worse in route learning (i.e., body-centered navigation) and had a tendency to perform worse in some aspects of wayfinding (i.e., world-centered navigation) than the non-AD aMCI participants. The participants with AD aMCI and non-AD aMCI performed similarly in conventional cognitive tests. The lower thickness of the parietal regions (i.e., the precuneus and the posterior parietal cortex) was associated with worse route learning performance. Lower volume of the MTL, especially the right posterior hippocampus and the pmEC, was associated with worse wayfinding performance. Lower thickness of the parietal regions and the right isthmus cingulate/RSC and lower volume of the MTL, especially the posterior hippocampus and the pmEC, were associated with worse performance in the perspective taking/wayfinding task. Lower levels of amyloid-β_1–42_ were associated with worse route learning performance, whereas higher levels of p-tau_181_ were associated with worse wayfinding performance and worse performance in the perspective taking/wayfinding task. Specifically, lower levels of amyloid-β_1–42_ were directly associated with worse route learning performance and this association was not mediated by the thickness of the parietal regions.

### Route-Repetition Task

#### Route-Repetition Task Performance Is Different in AD aMCI and Non-AD aMCI Participants

Consistent with our hypothesis, this study demonstrated that the aMCI participants with positive AD biomarkers showed worse route learning performance than the aMCI participants with negative AD biomarkers above and beyond demographic characteristics. The participants with AD aMCI and non-AD aMCI did not significantly differ in any conventional cognitive test. Next, the AD biomarker positive aMCI participants performed similarly to the participants with mild AD dementia, while the AD biomarker negative aMCI participants showed similar performance as the CN participants. These results support and further extend recent findings showing that testing of route learning in a large scale real environment can differentiate amyloid-β positive and negative patients with aMCI, where the latter did not differ from CN older adults (Schöberl et al., 2020). The results are also consistent with previous work indicating that body-centered direction estimation in a virtual reality navigation task can discriminate cognitively impaired older adults with AD from those with other neurodegenerative diseases (Tu et al., 2015, 2017). It should be noted that one study investigating spatial navigation in preclinical AD showed no differences in route learning between CN older adults with and without amyloid-β pathology, as measured by CSF amyloid-β_1–42_ levels (Allison et al., 2016). Together, these findings suggest that route learning deficits may be relatively specific to early clinical stages of AD but do not occur in the earliest, preclinical stage of AD. In accordance with our recent findings (Laczó et al., 2021a), results of the current study showed that all participants performed above the chance level and improved in route learning across all three experimental sessions indicating preserved learning and the absence of a floor effect in our Route-repetition task even in the participants with mild AD dementia.

#### Route-Repetition Task Performance Is Associated With Amyloid-β Pathology and Parietal Atrophy

Our results further demonstrated that greater amyloid-β pathology measured by amyloid-β_1–42_ levels in CSF was associated with worse route learning performance above and beyond demographic characteristics. This was consistent with our hypotheses and also with recent data showing that increased cortical amyloid-β accumulation relates to worse scene recognition from a constant first-person viewpoint in CN adults and patients with early AD (Maass et al., 2019). Our data also showed that cortical thinning in the precuneus and the posterior parietal cortex was associated with worse route learning performance above and beyond demographic characteristics. This was in accordance with our hypotheses and with the previously described important role of the parietal regions in route learning/body-centered navigation (Wolbers et al., 2004; Weniger et al., 2011). The finding indicates that impairment of this spatial navigation ability reflects neurodegeneration in the parietal cortex, which is typically found in early AD (Landau et al., 2011). Next, in agreement with our hypotheses, lower amyloid-β_1–42_ in CSF was associated with worse route learning directly and not indirectly through cortical thinning in the parietal regions when controlled for demographic characteristics. This indicates that both amyloid-β deposition and neurodegeneration in the parietal cortex may contribute to route learning deficits in early AD, where neurodegeneration may not be a direct result of amyloid-β accumulation. Amyloid-β accumulation in the parietal cortex that emerges very early in AD (Villain et al., 2012; Palmqvist et al., 2017) was shown to be related to less accurate spatial discrimination (Maass et al., 2019). It should be noted that CSF amyloid-β_1–42_ is a biomarker of amyloid-β pathology that is not specific to regional amyloid-β deposition (Palmqvist et al., 2015). Therefore, based on our data we could not demonstrate whether amyloid-β accumulation in the parietal cortex was associated with worse route learning performance. Future studies with amyloid PET quantifying regional-specific amyloid-β deposition are needed to explore this association.

#### The Association Between Route-Repetition Task Performance and Atrophy of the Right alEC

Contrary to our hypothesis, lower volume of the right alEC was associated with worse route learning, controlling for demographic characteristics. Rodent research showed that the lateral EC, a homolog of the human alEC, plays a role in processing spatial information in a body-centered frame of reference (Wang et al., 2018; Kuruvilla et al., 2020) and using proximal landmarks for navigation (Kuruvilla and Ainge, 2017). Next, a recent human study found that the alEC encodes distance information from landmarks (Chen et al., 2019). Our study may suggest that the alEC could also play a role in computing directional information from proximal landmarks. However, our finding should be interpreted with caution, because no association with the alEC was found for other tasks. Future studies are needed to investigate this potential association in detail.

### Route-Retracing Task

#### Route-Retracing Task Performance Is Impaired in AD aMCI and Non-AD aMCI Participants

The current study demonstrated that the aMCI participants with positive and negative AD biomarkers showed worse wayfinding performance in a virtual environment than the CN participants, above and beyond demographic characteristics. This is in agreement with our hypothesis and also with a recent finding showing wayfinding deficits in amyloid-β positive and negative patients with aMCI in real space (Schöberl et al., 2020). Contrary to our hypothesis, our results did not show significant overall differences in wayfinding performance between the aMCI participants with positive and negative AD biomarkers. However, analysis of differences from chance performance suggested that the aMCI participants with positive and negative AD biomarkers may have a different pattern of wayfinding deficits. Specifically, the AD biomarker positive aMCI participants performed at the chance level in the first two of three sessions, whereas the AD biomarker negative aMCI participants performed above the chance level across all sessions. Performance at the chance level in the aMCI participants with positive AD biomarkers also suggested that there may be a floor effect in the Route-retracing task leading to the non-significant differences between AD biomarker positive and negative aMCI participants in the main analysis. Furthermore, the *post-hoc* analysis showed that the aMCI participants with positive AD biomarkers performed worse than the aMCI participants with negative AD biomarkers in the second session of the task. However, this result should be interpreted with caution as there was no significant interaction between group and session. Together, these results indicated that aMCI participants with positive and negative AD biomarkers have wayfinding deficits that tend to be more pronounced in the former group. Wayfinding testing has previously reliably identified individuals with preclinical AD among CN older adults (Allison et al., 2016, 2019). However, previous studies examining wayfinding/world-centered navigation testing as a promising tool to differentiate cognitively impaired individuals with AD and those with other pathologies yielded inconsistent results. A recent study showed that wayfinding performance can reliably discriminate amyloid-β positive and negative aMCI patients in a navigation test in a large scale real environment that required the planning of novel routes including shortcuts (Schöberl et al., 2020). In a virtual reality navigation task, identification of the correct location on a map that required world-centered spatial information did not discriminate between cognitively impaired older adults with AD and those with other neurodegenerative diseases, whose performance was similarly worse than that of age-matched healthy controls (Tu et al., 2017). Overall, studies suggested that preclinical AD is characterized by wayfinding deficits (Allison et al., 2016, 2019) that worsen with disease progression to aMCI and dementia (Hort et al., 2007; Levine et al., 2020). However, wayfinding deficits are also found in cognitively impaired older adults without AD pathology (Tu et al., 2017; Schöberl et al., 2020), reducing the usefulness of wayfinding/world-centered navigation tasks for discriminating aMCI individuals with positive and negative AD biomarkers. The ability of these tasks to differentiate AD biomarker positive and negative older adults with cognitive impairment may also depend on their specific characteristics. These characteristics include the complexity of the task, whether there is a floor effect in the task, whether the task requires planning of new routes, and potentially also whether the navigation task takes place in real space or in virtual environment.

#### Route-Retracing Task Performance Is Associated With Atrophy of the Posterior MTL Subregions

Consistent with our hypothesis, lower volumes of the right posterior hippocampus (i.e., the hippocampal body) and the pmEC were associated with worse wayfinding performance above and beyond demographic characteristics. Previous studies showed that the right hippocampus is more strongly associated with wayfinding than the left hippocampus (Maguire et al., 1998; Nedelska et al., 2012; Laczó et al., 2017). In particular, its posterior subregion is involved in the accurate creation and use of cognitive maps (Doeller et al., 2008; Schinazi et al., 2013). The pmEC is important for world-centered direction computations (Chadwick et al., 2015) and spatial information processing (Berron et al., 2018). Our results are consistent with these findings and indicate that wayfinding deficits reflect neurodegeneration in the posterior MTL subregions in older adults. Neurodegeneration in the MTL including the hippocampus and the EC is a common finding in early AD (Scahill et al., 2002; Du et al., 2004; Tapiola et al., 2008) but is also observed in other neurodegenerative diseases (Jack et al., 2002; Nelson et al., 2019). In the current study, smaller MTL volumes were observed predominantly in the posterior (i.e., the hippocampal tail, right hippocampal body, and the pmEC) than in the anterior (i.e., the right alEC) subregions in the aMCI participants with positive AD biomarkers when compared to those with negative AD biomarkers. This supports previous work showing that in AD, compared to other neurodegenerative diseases, atrophy is more pronounced in the posterior MTL subregions (Lindberg et al., 2017; Lladó et al., 2018).

#### Route-Retracing Task Performance Is Associated With Tau Pathology

Consistent with our hypothesis, higher p-tau_181_ was associated with worse wayfinding performance, above and beyond demographic characteristics. Thus, our findings suggest that tau pathology may contribute to wayfinding deficits in cognitively impaired older adults. This is consistent with rodent data showing that in older mice, tau pathology is related to world-centered spatial memory deficits (Fu et al., 2017). It may also complement recent findings in CN older adults on the association between higher p-tau_181_ in CSF and worse wayfinding performance in a virtual environment (Allison et al., 2019). However, it is noteworthy that the previous study also found an association between amyloid-β_1–42_ in CSF and wayfinding performance in CN older adults (Allison et al., 2019). Tau pathology in the hippocampus and the EC (Braak and Braak, 1995) together with neocortical amyloid-β deposition (Hyman et al., 2012) is considered a major pathological marker of AD. A similar pattern of regional tau deposition without amyloid-β pathology has been observed in other neurodegenerative diseases including primary age-related tauopathy (Crary et al., 2014) and argyrophilic grain disease (Ferrer et al., 2008). Thus, unlike the association between amyloid-β pathology and route learning, the association between tau pathology and wayfinding may not be specific to AD. Previous work (Jacobs et al., 2018) showed that the spread of tau pathology to the posterior regions, which is facilitated by amyloid-β deposition, contributes to region-specific neurodegeneration, which in turn leads to cognitive impairment. Based on these findings, one would expect that tau pathology should be related to atrophy of the posterior MTL subregions, which in turn should be associated with wayfinding deficits. However, contrary to our hypothesis, we found that higher p-tau_181_ in CSF was associated with worse wayfinding directly and not indirectly through volume reduction in the posterior MTL (i.e., the pmEC), above and beyond demographic characteristics. This result is most likely driven by a non-significant relationship between p-tau_181_ levels in CSF and region-specific brain atrophy in our sample. However, this finding should be interpreted with caution as our results are based on a subsample of participants with CSF data. Thus, the analyses might not have sufficient power to demonstrate a significant association of p-tau_181_ in CSF with region-specific volume reduction, and the mediation effect of posterior MTL atrophy in the association between p-tau_181_ and wayfinding. It should be noted that CSF p-tau_181_ is a biomarker of tau pathology that is not specific to regional tau deposition (Schöll et al., 2019). Also, tau accumulation, measured by tau PET, was shown to be more strongly associated with neurodegeneration and cognitive decline than p-tau_181_ in CSF (Mattsson et al., 2017). Specifically, tau pathology in the MTL subregions, particularly in the posterior EC, was related to EC atrophy and mirrored its association with memory decline (Maass et al., 2018). Therefore, future studies with tau PET ligands are needed to explore in detail the association between regional-specific tau pathology, neurodegeneration in the posterior MTL subregions, and wayfinding in older adults.

### Directional-Approach Task

#### Directional-Approach Task Performance Is Impaired in AD aMCI and Non-AD aMCI Participants

In the third spatial navigation task, we assessed perspective taking and wayfinding. The task required participants to use the configuration of landmarks at an intersection to interpret a spatial situation from a viewpoint they had not experienced before. Consistent with our hypothesis, we demonstrated that the aMCI participants with positive and negative AD biomarkers and the participants with mild AD dementia performed worse than the CN participants, above and beyond demographic characteristics. These results support and further extend our recent findings indicating that patients with aMCI and mild AD dementia, who were not defined by biomarkers, show spatial navigation impairment in the same task (Laczó et al., 2021a) and perspective taking deficits in a virtual arena task (Marková et al., 2015). The participants’ performance in the current task generally decreased when the approach direction in the test phase was misaligned with the encoding phase by 180° compared to 90°, thus when the perspective shift between encoding and retrieval was higher. This finding is consistent with earlier work showing that perspective taking is required to solve the task (de Condappa and Wiener, 2016; Laczó et al., 2021a). Contrary to our hypothesis, we did not find significant differences between the aMCI participants with positive and negative AD biomarkers. It is worth noting that the aMCI participants with positive and negative AD biomarkers performed at the chance level when the direction in the test phase was misaligned by 180°. This indicates that large misalignment placed great demands on perspective taking. The task could therefore be difficult for most cognitively impaired older adults, as our previous study showed (Laczó et al., 2021a), and may be prone to the floor effect. On the other hand, a recent study indicated that older adults with aMCI and positive CSF AD biomarkers had worse recognition of the topographical layout of four mountains within a computer-generated environment from a shifted point of view than those with negative CSF AD biomarkers (Chan et al., 2016). The discrepant findings in our study and the previous study may be due to the different nature and difficulty of both tasks. Specifically, the degree of misalignment between the encoding and the test perspective differed greatly between these studies. In the previous study (Chan et al., 2016), the degree of misalignment ranged between 15° and 90°, and in our current study, the perspective was misaligned by 90° and 180°. Therefore, the demands on perspective taking were considerably higher in our task, which could result in non-significant differences between the cognitively impaired participants.

#### Directional-Approach Task Performance Is Associated With Atrophy of the Posterior MTL Subregions and Parietal Cortex

Consistent with our hypothesis, lower volumes of the posterior MTL subregions, especially the posterior hippocampus (i.e., the right hippocampal tail and the left hippocampal body) and the pmEC, together with cortical thinning in the precuneus, the posterior parietal cortex, and the right isthmus cingulate/RSC were associated with worse performance in the perspective taking/wayfinding task, above and beyond demographic characteristics. Our findings, therefore, indicated that multiple specific brain regions are involved in perspective taking and wayfinding. This is in accordance with the previously reported important role of the MTL (Lambrey et al., 2008) and the parietal regions (Zacks and Michelon, 2005) in perspective taking, and the role of the posterior hippocampus (Schinazi et al., 2013) and the pmEC (Chadwick et al., 2015) in world-centered navigation. Our findings are also consistent with the notion that the RSC plays a key role in the use of landmarks for directional orientation (Auger et al., 2012) and the integration of body-centered and world-centered spatial information (Clark et al., 2018), both of which can be important for successful task performance. Our results further indicate that perspective taking/wayfinding deficits reflect neurodegeneration in the posterior MTL subregions, parietal cortex, and the isthmus cingulate/RSC in older adults. This supports and further extends recent findings showing that atrophy of the hippocampus and precuneus is associated with worse recognition of the computer-generated environment from a shifted viewpoint in cognitively impaired and CN older adults (Chan et al., 2016).

#### Directional-Approach Task Performance Is Predominantly Associated With Tau Pathology

In accordance with our hypothesis, higher levels of p-tau_181_ in CSF were associated with worse task performance, above and beyond demographic characteristics. Also consistent with our hypothesis, higher levels of total tau and lower levels of amyloid-β_1–42_ in CSF correlated with worse performance. However, these findings should be interpreted with caution as the associations of amyloid-β_1–42_ and total tau in CSF with task performance were not significant after controlling for demographic characteristics. Previous research showed that lower amyloid-β_1–42_ (of borderline significance) and higher total tau in CSF are associated with worse recognition of the computer-generated environment from a shifted viewpoint in patients with aMCI (Wood et al., 2016). Next, the recent study indicated that higher p-tau_181_ and lower amyloid-β_1–42_ in CSF are associated with worse wayfinding performance in a virtual environment in CN older adults (Allison et al., 2019). Overall, these findings, including ours, suggested that tau and amyloid-β pathologies may contribute to perspective taking/wayfinding deficits in older adults with and without cognitive impairment. In addition, they suggest that neurodegeneration (as measured by total tau in CSF) may contribute to perspective taking deficits in cognitively impaired older adults. It should be noted that the association between p-tau_181_ levels and task performance was not significant in the mediation analyses with MRI brain measures. Next, the association between amyloid-β_1–42_ levels and task performance was not significant in the regression analyses, controlling for demographic characteristics. Therefore, we were unable to support our hypotheses about the different mediation effects of regional brain atrophy in these associations. It is plausible that the analyses based on a subsample of participants with CSF data might not have sufficient power to demonstrate significant associations with perspective taking/wayfinding performance. Our preliminary findings should therefore be interpreted with caution and verified by future studies. Future studies with larger cohorts of participants are needed to explore in detail the interrelations between tau and amyloid-β pathologies (measured by CSF biomarkers and PET), regional brain atrophy, and perspective taking in cognitively impaired older adults.

### Characteristics of the Participants With Non-AD aMCI

The participants with non-AD aMCI were defined by negative amyloid-β biomarkers including normal CSF amyloid-β_1–42_ and negative amyloid PET imaging. However, the underlying disease causing their cognitive impairment was mostly unknown. It is worth noting that 60% of these participants with available CSF biomarkers had abnormal p-tau_181_ levels indicating that tau pathology was the underlying condition. About 60% of the participants with underlying tau pathology also had abnormal total tau levels indicative of neurodegeneration. This may at least partially explain non-significant differences between the participants with non-AD aMCI and AD aMCI in p-tau_181_ and total tau levels. However, it should be noted that the participants with non-AD aMCI had almost twice as low levels of p-tau181 and total tau compared to those with AD aMCI. As only 45% of the aMCI participants underwent CSF biomarker assessment, the non-significant differences between the non-AD aMCI and AD aMCI participants may have been due to the insufficient power of the analyses. The participants with non-AD aMCI did not show significant brain atrophy on MRI compared to the CN participants. However, we cannot rule out ongoing neurodegenerative diseases because FDG-PET, a sensitive marker of regional neurodegeneration, was not performed. The general uncertainty regarding the etiology of the non-AD aMCI participants is consistent with previous studies showing that this group may consist of various entities, including the neurodegenerative ones (i.e., tauopathy, synucleinopathy, and TDP-43 pathology), that may fully manifest several years later (Villemagne et al., 2011; Ye et al., 2018). Longitudinal follow-up is therefore essential to detect the underlying disease.

### Strengths and Limitations of the Study

One of the strengths of the current study is the fact that this is the first study to date to comprehensively examine the differences between AD biomarker positive and negative older adults with aMCI in various navigational abilities (i.e., route learning, wayfinding, and perspective taking) using an established and ecologically valid spatial navigation test in a realistic-looking virtual environment that mimics real-life navigation. In addition, we investigated the complex interrelations between structural measures of the specific MTL subregions, cortical and subcortical brain regions, CSF AD biomarkers, and spatial navigation that have not been thoroughly studied. Finally, we used well-defined homogeneous cohorts of CN participants and cognitively impaired older adults, where the diagnosis of AD and non-AD was supported by biomarker assessment including amyloid PET imaging and CSF biomarkers. There are also several limitations to this study. First, assessment of CSF biomarkers and amyloid PET imaging were not performed in the CN participants to rule out preclinical AD, which may limit the generalizability of our findings. However, it should be noted that we used several inclusion criteria to minimize the possibility of recruiting individuals at increased risk of AD. Second, CSF AD biomarkers were available in a subset of the participants (i.e., 47 of the 92 participants with cognitive impairment), which may reduce the statistical power of the observed associations with spatial navigation performance in the regression and mediation analyses. Therefore, these findings should be interpreted with caution and replicated in larger study cohorts. Third, the statistical power to detect significant differences with medium effect sizes for the interactions in the main analyses of spatial navigation performance was less than 0.80. Therefore, the results of the interactions, especially the non-significant ones, should be interpreted with caution. Fourth, amyloid-β_1–40_ was not available and therefore we could not use amyloid-β_1–42_/amyloid-β_1–40_ ratio as a more reliable marker of amyloid-β pathology. Fifth, the dichotomous visual read of amyloid PET did not allow quantifying amyloid-β accumulation in specific brain regions of interest and examining its association with spatial navigation performance, which should be the focus of future studies. Sixth, the underlying disease of the non-AD aMCI participants was mostly unknown, which may limit the interpretation and generalizability of our findings. Seventh, segmentation of the hippocampus and EC was performed on 3D T1w images, whereas the use of T2-weighted images would be more preferable as it could provide better resolution. Eight, because of the lack of an anatomical mask for measurement of the RSC, we used a proximal anatomical measure, the thickness of the isthmus cingulate, which may limit the specificity of our results and their interpretation regarding the role of the RSC in spatial navigation. Ninth, given that previous studies considered the subregions of the posterior hippocampus (i.e., body and tail) as a single region, and given the lack of specific hypotheses regarding their equal or different associations with spatial navigation performance, our results should be considered exploratory and these associations should be examined in more detail in future studies. Finally, the cross-sectional design did not allow evaluating changes in spatial navigation performance over time, but longitudinal follow-up is ongoing.

## Conclusions

Using the Navigation Test Suite, the current study demonstrated that older adults with aMCI and positive AD biomarkers showed worse spatial navigation performance compared to those with negative AD biomarkers although both groups performed similarly in conventional cognitive tests. The profile and severity of spatial navigation deficits in the AD aMCI and non-AD aMCI participants varied between the tasks. Specifically, route learning (i.e., body-centered navigation) was worse in the AD aMCI participants than in the non-AD aMCI participants who were similar to the CN older adults. Wayfinding (i.e., world-centered navigation) deficits were found in the AD aMCI and non-AD aMCI participants and tended to be more pronounced in those with AD aMCI. Spatial navigation deficits in the perspective-taking/wayfinding task were observed in aMCI participants regardless of the biomarker status. The Navigation Test Suite thus provides complex information about spatial navigation abilities in cognitively impaired older adults and may complement cognitive assessment with conventional neuropsychological tests. This test may also help distinguish cognitive deficits in early AD from those in other diseases. Further, we demonstrated that deficits in various spatial navigation abilities were differently associated with neurodegeneration in the MTL and posterior cortical regions, the areas that atrophy early during the AD progression. Route learning deficits were associated with parietal cortical atrophy and wayfinding deficits were associated with posterior MTL atrophy. Navigation deficits in the perspective taking/wayfinding task were associated with atrophy in several brain regions. Finally, we demonstrated that various spatial navigation abilities reflected different aspects of AD pathology as measured by CSF biomarkers. Specifically, route learning deficits were directly associated with amyloid-β pathology regardless of parietal atrophy and wayfinding deficits were associated with tau pathology. Navigation deficits in the perspective taking/wayfinding task were associated with tau pathology and also correlated with amyloid-β pathology and neurodegeneration measured by CSF total tau (without adjustment for demographic characteristics). In conclusion, the Navigation Test Suite has the potential to improve the early detection of AD-related cognitive deficits and could be used as a screening tool for the diagnosis of early AD in clinical settings. It should be noted that this test may have some limitations due to the floor effect in the wayfinding and perspective taking/wayfinding tasks that deserve further attention. The search for new reliable cognitive screening tools is of great importance as PET imaging, CSF, and blood-based biomarkers are currently limited to research settings and expert clinics. This is also particularly important as therapeutic interventions targeting the AD pathology are now becoming available.

## Data Availability Statement

The datasets presented in this article are not readily available because of the policy of the Czech Brain Aging Study (CBAS), which allows sharing of the data only after previous approval. Requests to access the datasets should be directed to JL, jan.laczo@lfmotol.cuni.cz.

## Ethics Statement

The studies involving human participants were reviewed and approved by EK - 701/16 25.5.2016. The participants provided their written informed consent to participate in this study.

## Author Contributions

ML and JL participated in the design of the study, data interpretation, and wrote the draft of the manuscript. JW participated in the design of the study. LM, OL, and ZN were involved in MRI data acquisition and processing. JK, VM, MV, and JH were involved in data acquisition and interpretation. All authors contributed to the article and approved the submitted version.

## Conflict of Interest

The authors declare that the research was conducted in the absence of any commercial or financial relationships that could be construed as a potential conflict of interest.

## Publisher’s Note

All claims expressed in this article are solely those of the authors and do not necessarily represent those of their affiliated organizations, or those of the publisher, the editors and the reviewers. Any product that may be evaluated in this article, or claim that may be made by its manufacturer, is not guaranteed or endorsed by the publisher.

## Footnotes

^1^ https://osf.io/mx52y/

^2^ http://stnava.github.io/ANTs/

^3^ http://dbm.neuro.uni-jena.de/vbm/
