# Supplementary material for: Different Profiles of Spatial Navigation Deficits In Alzheimer’s Disease Biomarker-Positive Versus Biomarker-Negative Older Adults With Amnestic Mild Cognitive Impairment
Source: Front Aging Neurosci. 2022 Jun 2;14:886778. doi: 10.3389/fnagi.2022.886778 (PMC9201637; doi:10.3389/fnagi.2022.886778)
Supplement: Supplementary file 1 [file Data_Sheet_1.doc]

**Supplementary Table 1**  Characteristics of the participants with MRI brain data.

|  | **CN**  **(n = 29)** | **Non-AD aMCI**  **(n = 23)** | **AD aMCI**  **(n = 26)** | **Mild AD dementia**  **(n = 22)** | ***P* values** | **Effect sizes** |
| --- | --- | --- | --- | --- | --- | --- |
| Age (years) | 68.59 (5.87) *^c^ | 68.74 (7.34)*^c^ | 71.77 (7.02) | 74.05 (5.15) | .010 | 0.11 |
| Women, n (%) | 21 (72) | 13 (57) | 15 (58) | 16 (73) | .455 | 0.16 |
| Education (years) | 16.21 (1.80)***^a^ | 13.43 (2.15) | 15.19 (3.02) | 14.59 (3.02) | .001 | 0.15 |
| MMSE | 29.38 (0.90)**^a^, ***^b,c^ | 27.30 (2.29) ***^c^ | 26.00 (2.33)***^c^ | 21.95 (2.55) | <.001 | 0.64 |

Demographic and cognitive characteristics. Values are mean (SD) except for gender. *P* values refer to the main effect across all groups. *P* values indicate the level of significance *p < 0.05; **p < 0.01; ***p < 0.001; Effect sizes were calculated as Cramér’s V for the χ^2^ test (gender) and partial eta-squared for one-way analysis of variance (all other variables).

^a^Differences compared to the non-AD aMCI group.

^b^Differences compared to the AD aMCI group.

^c^Differences compared to the mild AD dementia group.

**Key:** MRI, magnetic resonance imaging; CN, cognitively normal; non-AD aMCI, amnestic mild cognitive impairment with negative AD biomarkers; AD aMCI, amnestic mild cognitive impairment with Alzheimer’s disease; mild AD dementia, mild dementia with Alzheimer’s disease; MMSE, Mini-Mental State Examination.

**Supplementary Table 2**  Characteristics of the participants with CSF data.

|  | **Non-AD aMCI**  **(n = 12)** | **AD aMCI**  **(n = 17)** | **Mild AD dementia**  **(n = 18)** | ***P* values** | **Effect sizes** |
| --- | --- | --- | --- | --- | --- |
| Age (years) | 67.75 (9.28) | 70.94 (5.04) | 73.83 (6.25) | .064 | 0.12 |
| Women, n (%) | 5 (42) | 9 (53) | 12 (67) | .390 | 0.20 |
| Education (years) | 13.67 (1.61) | 15.82 (2.92) | 14.89 (2.68) | .093 | 0.10 |
| MMSE | 26.83 (2.52) ***^c^ | 26.24 (1.72)***^c^ | 21.50 (2.55) | <.001 | 0.55 |

Demographic and cognitive characteristics. Values are mean (SD) except for gender. *P* values refer to the main effect across all groups. *P* values indicate the level of significance ***p < 0.001; Effect sizes were calculated as Cramér’s V for the χ^2^ test (gender) and partial eta-squared for one-way analysis of variance (all other variables).

^c^Differences compared to the mild AD dementia group.

**Key:** CSF, cerebrospinal fluid; non-AD aMCI, amnestic mild cognitive impairment with negative AD biomarkers; AD aMCI, amnestic mild cognitive impairment with Alzheimer’s disease; mild AD dementia, mild dementia with Alzheimer’s disease; MMSE, Mini-Mental State Examination.

**Supplementary Table 3**  Sensitivity and specificity for the relevant cut-off values for the Route-repetition task, Route-retracing task and Directional-approach task.

|  | **Cut-off value (%)** | **Sensitivity/Specificity (%)** |
| --- | --- | --- |
| **Route-repetition task** | | |
| CN vs. non-AD aMCI | 83 | 67/65 |
| CN vs. AD aMCI | 70 | 87/79 |
| CN vs. mild AD dementia | 70 | 87/82 |
| Non-AD aMCI vs. AD aMCI | 63 | 68/70 |
| Non-AD aMCI vs. mild AD dementia | 63 | 68/71 |
| AD aMCI vs. mild AD dementia | NA | NA |
| **Route-retracing task** | | |
| CN vs. non-AD aMCI | 57 | 73/60 |
| CN vs. AD aMCI | 50 | 80/73 |
| CN vs. mild AD dementia | 50 | 80/71 |
| Non-AD aMCI vs. AD aMCI | 43 | 67/52 |
| Non-AD aMCI vs. mild AD dementia | 43 | 67/57 |
| AD aMCI vs. mild AD dementia | NA | NA |
| **Directional-approach task** | | |
| CN vs. non-AD aMCI | 57 | 80/60 |
| CN vs. AD aMCI | 57 | 80/85 |
| CN vs. mild AD dementia | 57 | 80/93 |
| Non-AD aMCI vs. AD aMCI | NA | NA |
| Non-AD aMCI vs. mild AD dementia | 43 | 57/75 |
| AD aMCI vs. mild AD dementia | NA | NA |

**Key:** CN, cognitively normal; non-AD aMCI, amnestic mild cognitive impairment with negative AD biomarkers; AD aMCI, amnestic mild cognitive impairment with Alzheimer’s disease; mild AD dementia, mild dementia with Alzheimer’s disease.

**Supplementary Table 4**  Mediation analysis of the associations between CSF AD biomarkers, spatial navigation performance and MRI brain measures.

| **Independent variable** | **Dependent variable** | **Moderator** | **a path** | **b path** | **a*b path** | **c´ path** | **c path** |
| --- | --- | --- | --- | --- | --- | --- | --- |
| Amyloid-β_1-42_ | Route-repetition task | Precuneus left | 0.190 | 0.258 | 0.049 | 0.277* | 0.326* |
|  |  | Posterior parietal cortex right | 0.222 | 0.113 | 0.025 | 0.300* | 0.326* |
|  |  | Posterior parietal cortex left | 0.196 | 0.172 | 0.034 | 0.292* | 0.326* |
|  |  | alEC right | 0.371** | 0.223 | 0.083 | 0.243 | 0.326* |
|  |  | Precuneus right | 0.169 | 0.133 | 0.023 | 0.303* | 0.326* |
| P-tau_181_ | Route-retracing task | Hippocampal body right | -0.225 | 0.229 | -0.051 | -0.304 | -0.355* |
|  |  | pmEC right | -0.030 | 0.157 | -0.005 | -0.350* | -0.355* |
|  |  | pmEC left | 0.048 | 0.243 | 0.012 | -0.367* | -0.355* |
| P-tau_181_ | Directional-approach task | Hippocampal body left | -0.238 | 0.253 | -0.060 | -0.216 | -0.276 |
|  |  | Hippocampal tail right | -0.235 | 0.291 | -0.068 | -0.208 | -0.276 |
|  |  | pmEC left | 0.048 | 0.330 | 0.016 | -0.292* | -0.276 |
|  |  | Precuneus left | -0.401* | 0.284 | -0.040 | -0.236 | -0.276 |
|  |  | Precuneus right | -0.082 | 0.282 | -0.023 | -0.253 | -0.276 |
|  |  | pmEC right | -0.030 | 0.070 | -0.002 | -0.274 | -0.276 |
|  |  | Posterior parietal cortex left | -0.084 | 0.181 | -0.015 | -0.261 | -0.276 |
|  |  | Posterior parietal cortex right | -0.083 | 0.217 | -0.018 | -0.258 | -0.276 |
|  |  | Isthmus cingulate right | -0.176 | 0.191 | -0.034 | -0.242 | -0.276 |

**Key:** CSF, cerebrospinal fluid; AD, Alzheimer’s disease; MRI, magnetic resonance imaging; a path, effect of the independent variable on the moderator; b path, effect of the moderator on the dependent variable; a*b path, indirect effect of the independent variable on the dependent variable through the moderator; c’ path, direct effect of the independent variable on the dependent variable controlled for the mediator; c path, total effect of the independent variable on the dependent variable; p-tau_181_, phosphorylated tau_181_; alEC, anterolateral entorhinal cortex; pmEC, posteromedial entorhinal cortex.

**Supplementary table 5**  Correlation matrix of spatial navigation performance and MRI brain measures in the participants with CSF data.

|  | Route-repetition task (% correct) | Route-retracing task (% correct) | Directional-approach task (% correct) |
| --- | --- | --- | --- |
| Hippocampal head right^a^ (volume, cm^3^) | .272 | .232 | .146 |
| Hippocampal head left^a^ (volume, cm^3^) | .202 | .146 | .091 |
| Hippocampal body right^a^ (volume, cm^3^) | .364* | .441** | .436** |
| Hippocampal body left^a^ (volume, cm^3^) | .246 | .308 | .394* |
| Hippocampal tail right^a^ (volume, cm^3^) | .322* | .451** | .465** |
| Hippocampal tail left^a^ (volume, cm^3^) | .315* | .393* | .451** |
| alEC right^a^ (volume, cm^3^) | .502*** | .322* | .400* |
| alEC left^a^ (volume, cm^3^) | .355* | .108 | .290 |
| pmEC right^a^ (volume, cm^3^) | .369* | .329* | .321* |
| pmEC left^a^ (volume, cm^3^) | .535*** | .374* | .451** |
| Caudate nucleus right^a^ (volume, cm^3^) | .066 | .069 | .032 |
| Caudate nucleus left^a^ (volume, cm^3^) | -.016 | -.010 | -.076 |
| Precuneus right (thickness, mm) | .351* | .110 | .418** |
| Precuneus left (thickness, mm) | .449** | .203 | .430** |
| Isthmus cingulate right (thickness, mm) | .166 | .147 | .357* |
| Isthmus cingulate left (thickness, mm) | .051 | .032 | .058 |
| Posterior parietal cortex right (thickness, mm) | .361* | .168 | .361* |
| Posterior parietal cortex left (thickness, mm) | .385* | .162 | .341* |

* Correlation (uncorrected) is significant at the .05 level (2-tailed), ** Correlation (uncorrected) is significant at the .01 level (2-tailed), *** Correlation (uncorrected) is significant at the .001 level (2-tailed).

^a^Volume normalized to estimated total intracranial volume.

**Key:** MRI, magnetic resonance imaging; CSF, cerebrospinal fluid; alEC, anterolateral entorhinal cortex; pmEC, posteromedial entorhinal cortex.

**Supplementary table 6** Correlation matrix of CSF biomarkers and MRI brain measures in the participants with CSF data.

|  | Amyloid-β_1-42_ (pg/ml) | Total tau (pg/ml) | P-tau_181_ (pg/ml) |
| --- | --- | --- | --- |
| Hippocampal head right^a^ (volume, cm^3^) | .233 | -.394* | -.104 |
| Hippocampal head left^a^ (volume, cm^3^) | .313* | -.309 | -.068 |
| Hippocampal body right^a^ (volume, cm^3^) | .428** | -.518** | -.296 |
| Hippocampal body left^a^ (volume, cm^3^) | .343* | -.309 | -.297 |
| Hippocampal tail right^a^ (volume, cm^3^) | .504*** | -.511** | -.281 |
| Hippocampal tail left^a^ (volume, cm^3^) | .537*** | -.388* | -.275 |
| alEC right^a^ (volume, cm^3^) | .501*** | -.394* | -.218 |
| alEC left^a^ (volume, cm^3^) | .254 | -.148 | .078 |
| pmEC right^a^ (volume, cm^3^) | .359* | -.359* | -.110 |
| pmEC left^a^ (volume, cm^3^) | .384* | -.217 | .005 |
| Caudate nucleus right^a^ (volume, cm^3^) | .067 | -.312 | .028 |
| Caudate nucleus left^a^ (volume, cm^3^) | .003 | -.249 | -.084 |
| Precuneus right (thickness, mm) | .270 | -.298 | -.145 |
| Precuneus left (thickness, mm) | .295 | -.262 | -.206 |
| Isthmus cingulate right (thickness, mm) | .322* | -.241 | -.223 |
| Isthmus cingulate left (thickness, mm) | .306 | -.254 | -.324* |
| Posterior parietal cortex right (thickness, mm) | .337* | -.329* | -.150 |
| Posterior parietal cortex left (thickness, mm) | .275 | -.233 | -.145 |

* Correlation (uncorrected) is significant at the .05 level (2-tailed), ** Correlation (uncorrected) is significant at the .01 level (2-tailed), *** Correlation (uncorrected) is significant at the .001 level (2-tailed).

^a^Volume normalized to estimated total intracranial volume.

**Key:** CSF, cerebrospinal fluid; MRI, magnetic resonance imaging; alEC, anterolateral entorhinal cortex; pmEC, posteromedial entorhinal cortex.

**Supplementary Figure 1**  Significant correlations between spatial navigation performance and MRI brain measures.

**A.**
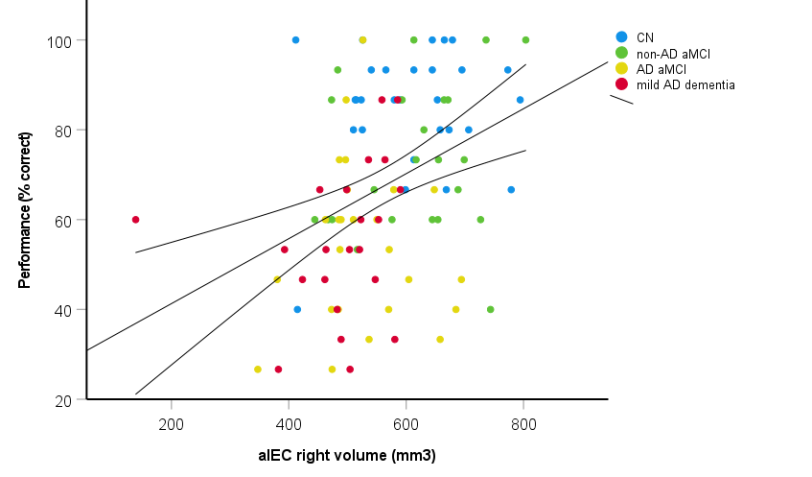
**Route-repetition task and MRI brain measures**

**
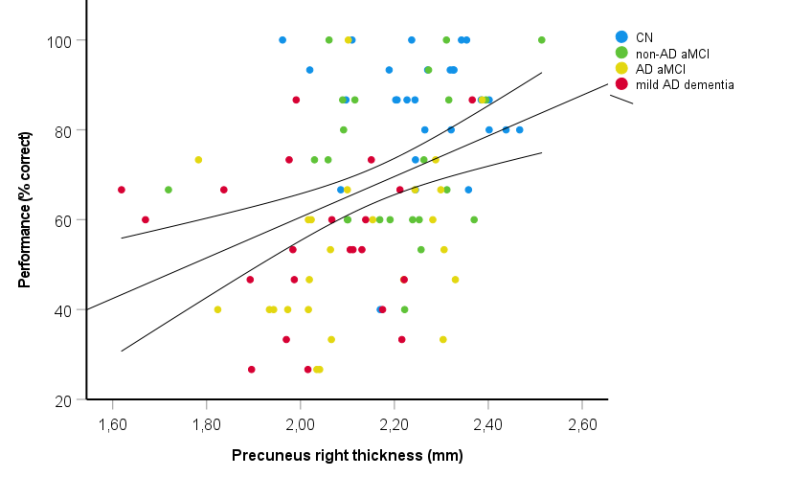
**

r = .374

p < .001

r = .380

p < .001


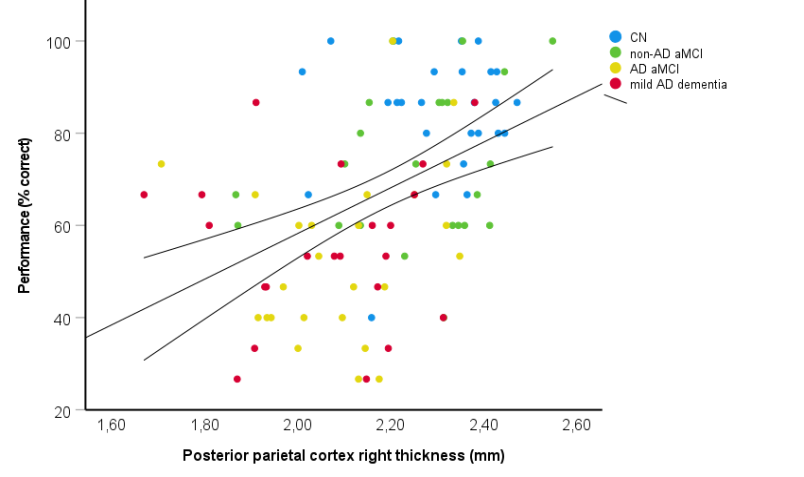


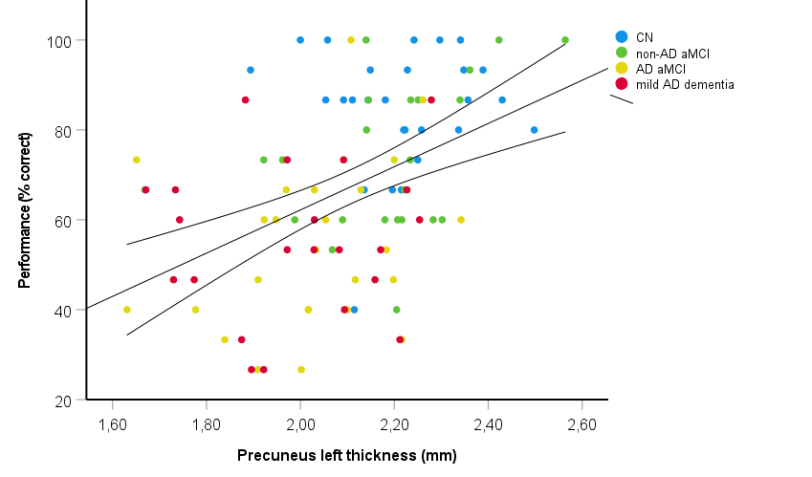


r = .446

p < .001

r = .444

p < .001


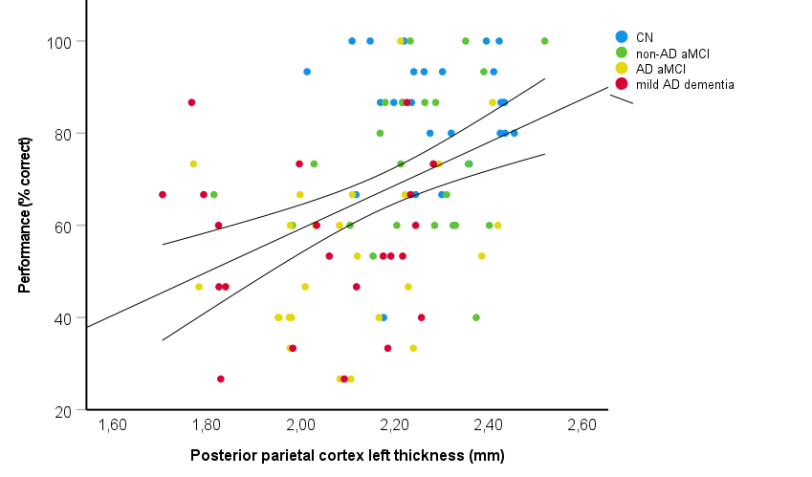


r = .419

p < .001

**B. Route-retracing task and MRI brain measures**


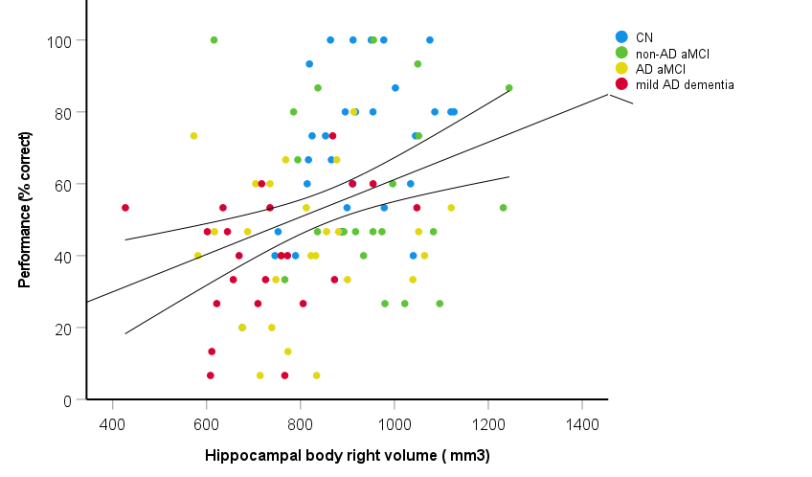

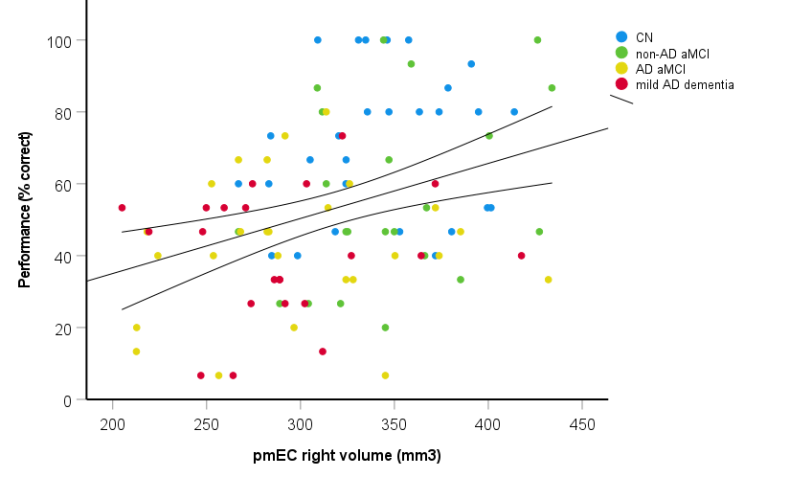


r = .345

p < .001

r = .344

p < .001

**Supplementary Fig 2.** Significant correlations between spatial navigation performance and CSF


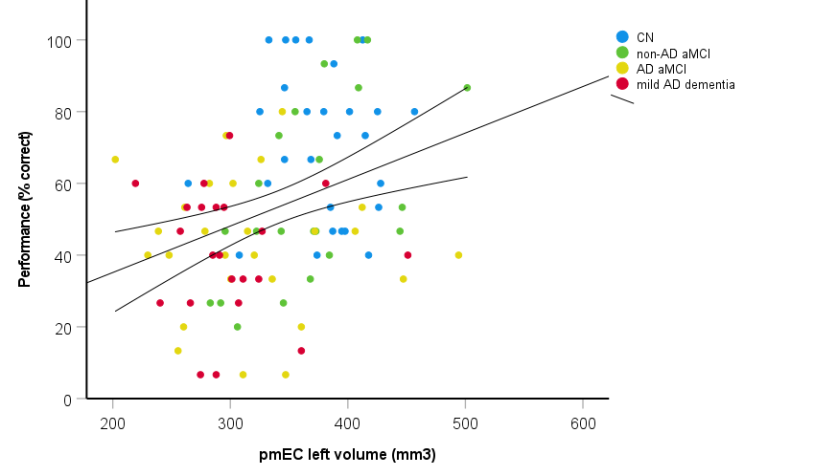


r = .340

p < .001


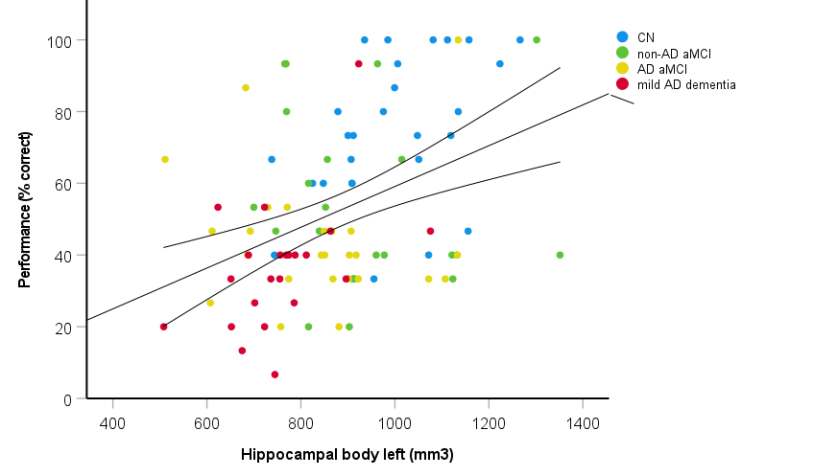

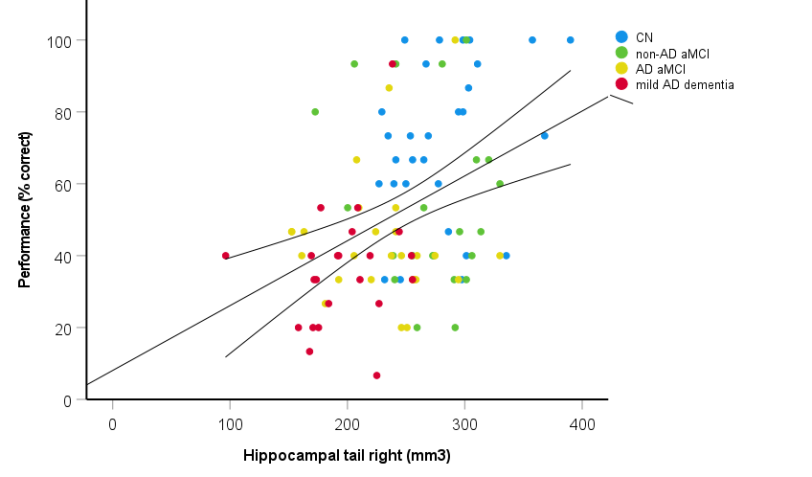
**C. Directional-approach task and MRI brain measures**

r = .397

p < .001

r = .392

p < .001


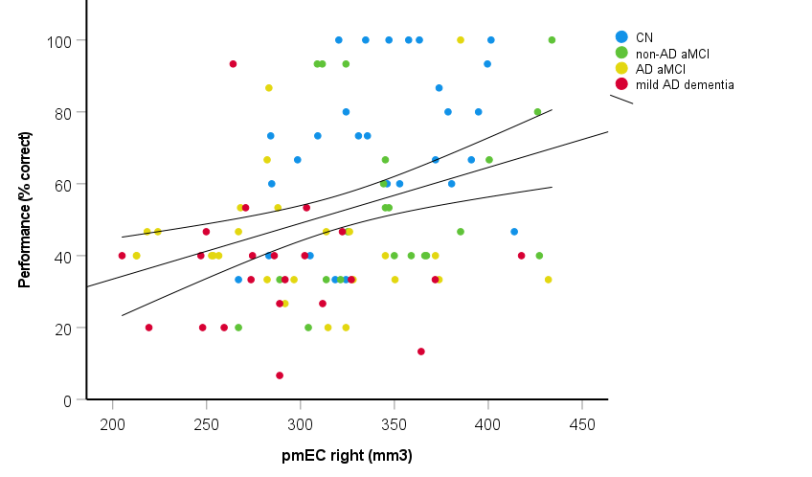

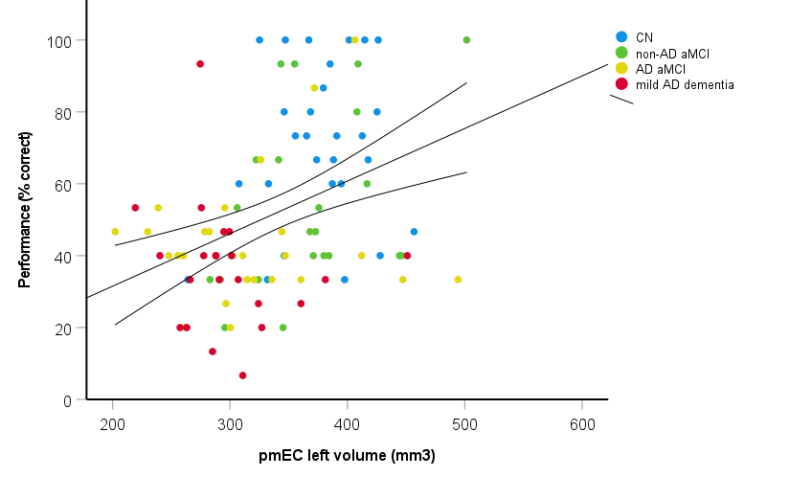


r = .345

p < .001

r = .397

p < .001


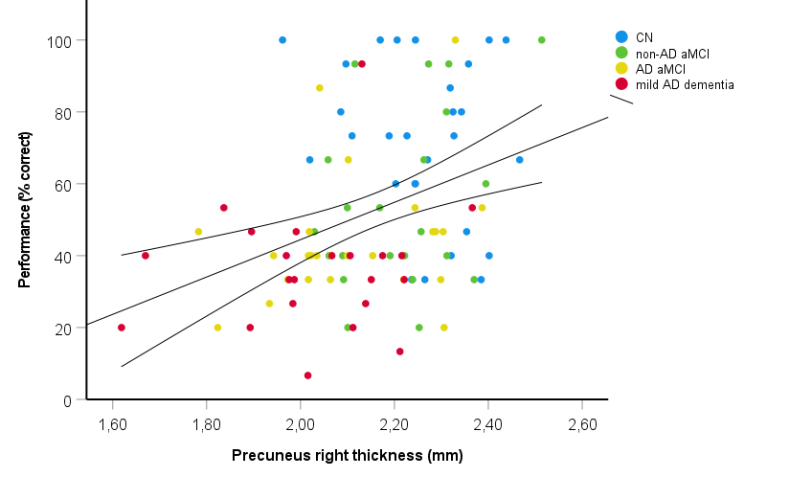

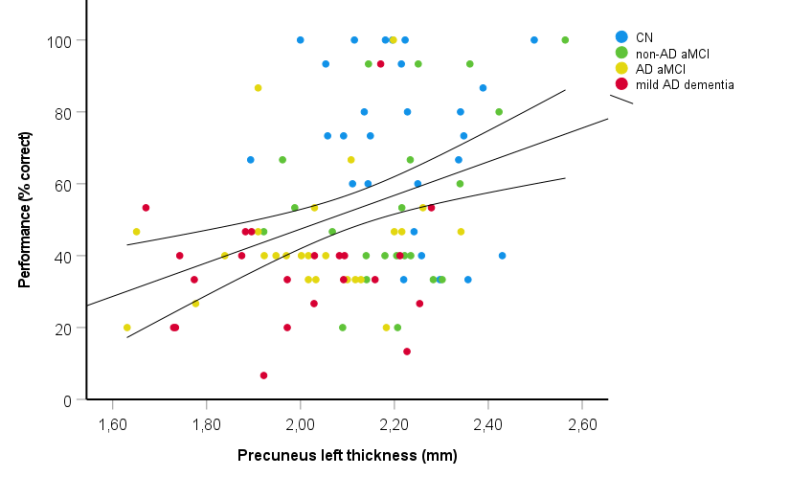


r = .361

p < .001

r = .359

p < .001


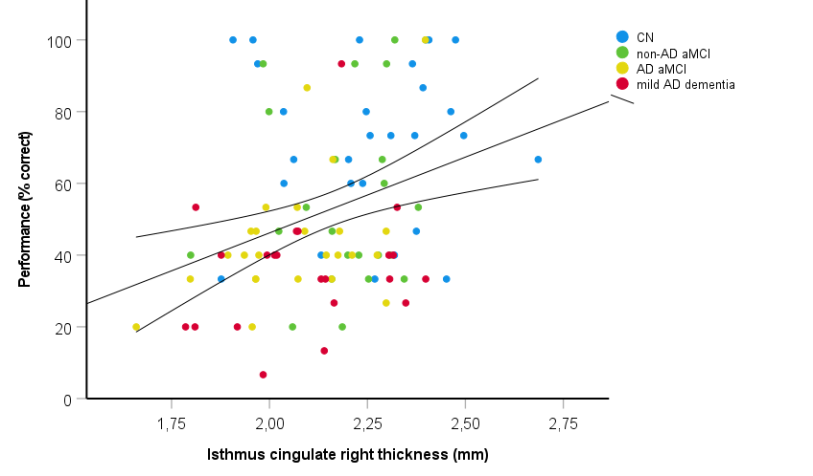

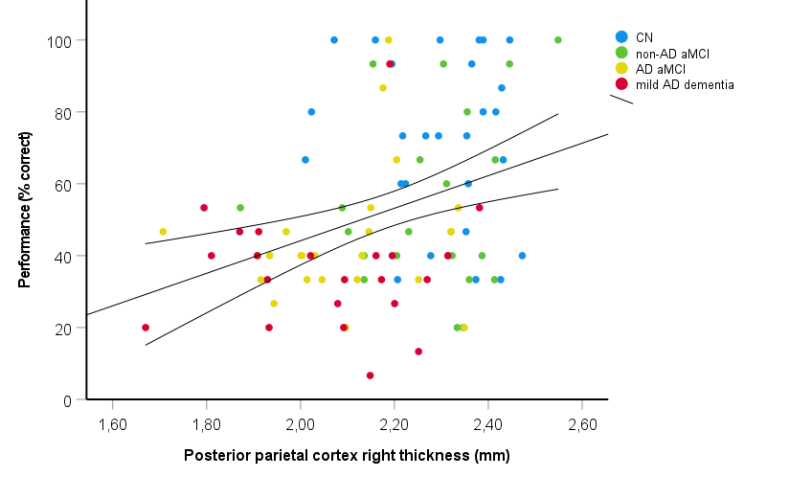


r = .326

p = .001

r = .339

p < .001


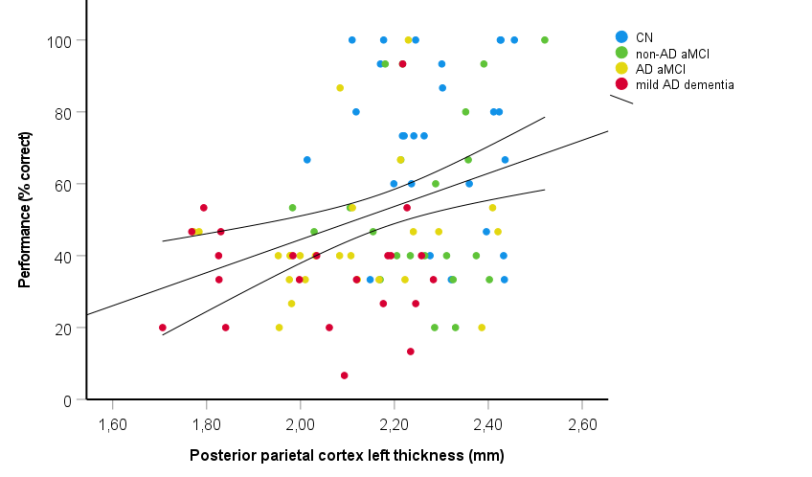


r = .342

p < .001

**Key:** MRI, magnetic resonance imaging; CN, cognitively normal; non-AD aMCI, amnestic mild cognitive impairment with negative AD biomarkers; AD aMCI, amnestic mild cognitive impairment with Alzheimer’s disease; mild AD dementia, mild dementia with Alzheimer’s disease; alEC, anterolateral entorhinal cortex; pmEC, posteromedial entorhinal cortex.

**Supplementary Figure 2**  Significant correlations between spatial navigation performance and CSF biomarkers.

**A. Route-repetition task and CSF measures**

**
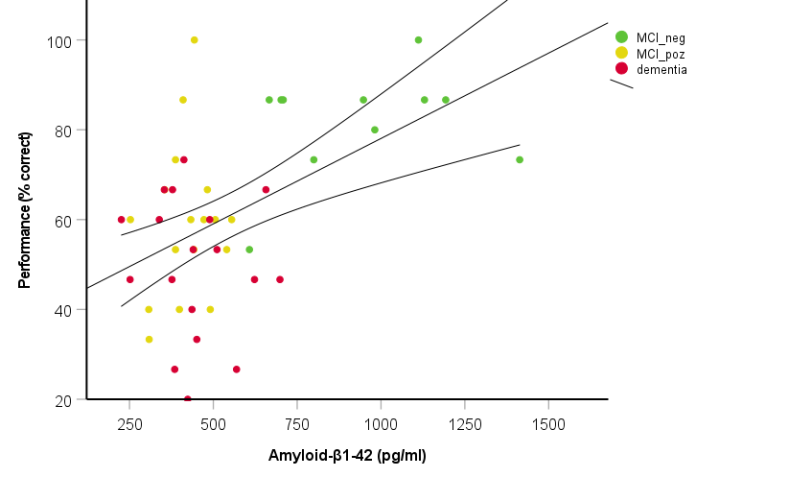
**

r = .425

p = .003

**B. Route-retracing task and CSF measures**

**
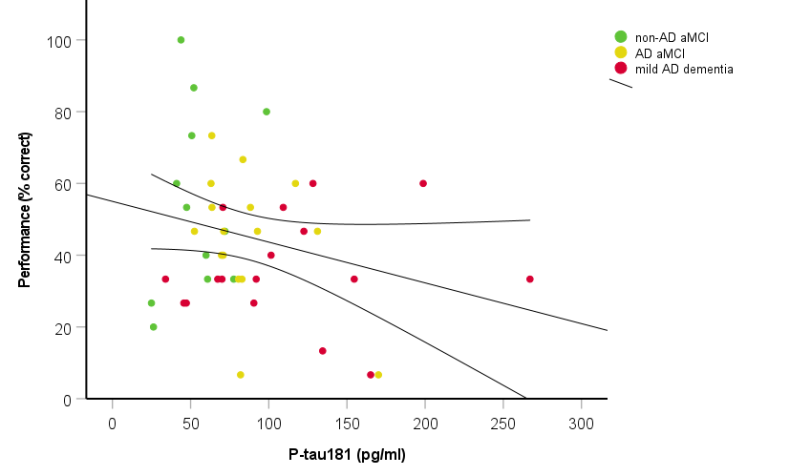
**

r = -.296

p = .043


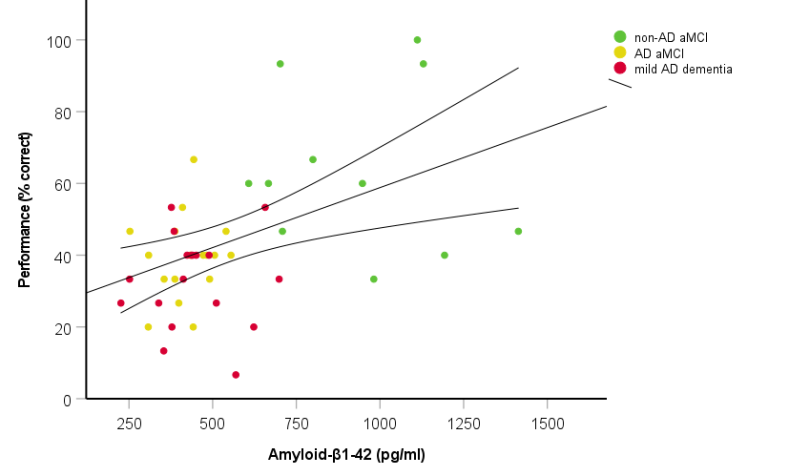

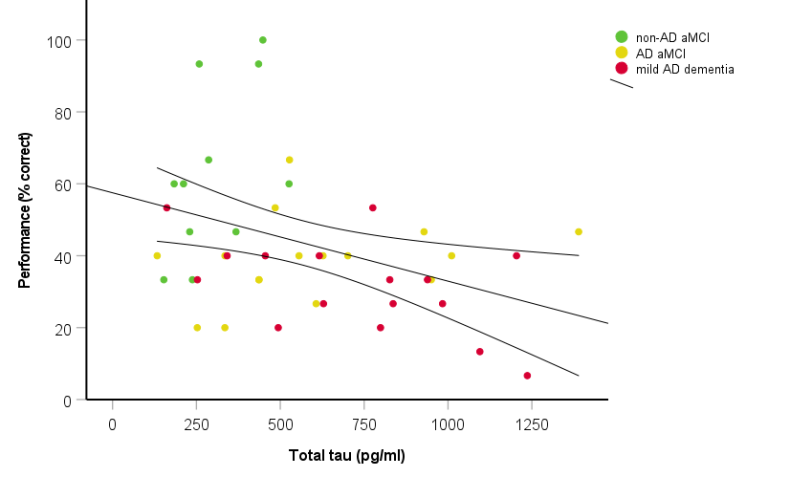
**C. Directional-approach task and CSF measures**

r = -.307

p = .041

r = .310

p = .032


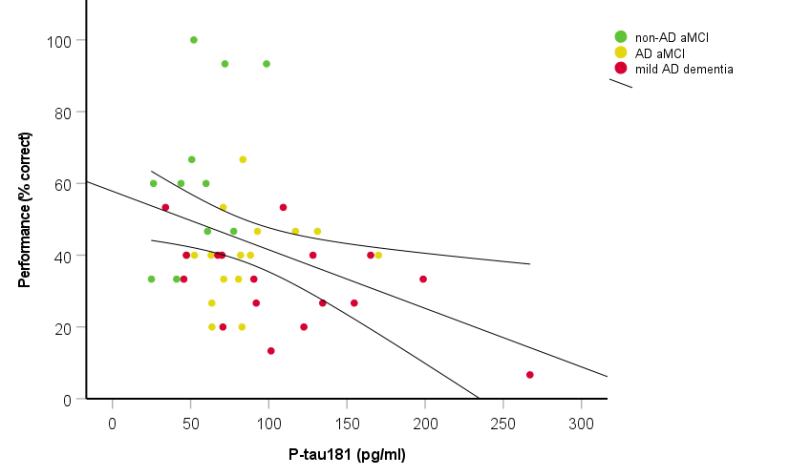


r = -.329

p = .024

Key: CSF, cerebrospinal fluid; non-AD aMCI, amnestic mild cognitive impairment with negative AD biomarkers; AD aMCI, amnestic mild cognitive impairment with Alzheimer’s disease; mild AD dementia, mild dementia with Alzheimer’s disease; p-tau_181_, phosphorylated tau_181_.
